# The Influence of Flavonoids with -Br, -Cl Atoms and -NO_2_, -CH_3_ Groups on the Growth Kinetics and the Number of Pathogenic and Probiotic Microorganisms

**DOI:** 10.3390/ijms25179269

**Published:** 2024-08-27

**Authors:** Martyna Perz, Daria Szymanowska, Edyta Kostrzewa-Susłow

**Affiliations:** 1 Department of Food Chemistry and Biocatalysis, Faculty of Biotechnology and Food Science, Wrocław University of Environmental and Life Sciences, 50-375 Wrocław, Poland; 2 Department of Biotechnology and Food Microbiology, Faculty of Food Science and Nutrition, Poznań University of Life Sciences, 60-627 Poznań, Poland; daria.szymanowska@up.poznan.pl; 3 Department of Pharmacognosy and Biomaterials, Faculty of Pharmacy, Poznań University of Medical Sciences, 60-806 Poznań, Poland

**Keywords:** flavonoids, antimicrobial properties, pathogenic microbes, probiotic bacteria, digestion in vitro, microbiome, halogens, nitro group, methyl group

## Abstract

The pursuit of novel or modified substances based on a natural origin, like flavonoids, is essential in addressing the increasing number of diseases and bacterial resistance to antibiotics, as well as in maintaining intestinal balance and enhancing overall gut health. The primary goal of this research was to evaluate the impact of specific flavonoid compounds—chalcones, flavanones, and flavones—substituted with -Br, -Cl, -CH_3_, and -NO_2_ on both pathogenic and probiotic microorganisms. Additionally, this study aimed to understand these compounds’ influence on standardized normal and pathologically altered intestinal microbiomes. 8-Bromo-6-chloroflavone 4′-*O*-*β*-D-(4″-*O*-methyl)-glucopyranoside and 8-bromo-6-chloroflavanone showed the most promising results as bactericidal agents. They significantly limited or inhibited the growth of pathogenic bacteria without adversely affecting the probiotic’s growth. Digestion in vitro studies indicated that 6-methyl-8-nitroflavone and 8-bromo-6-chloroflavone positively modulated the gut microbiome by increasing beneficial bacteria and reducing potentially pathogenic microbes. This effect was most notable in microbiomes characteristic of older individuals and those recovering from chemotherapy or antibiotic treatments. This study underscores the therapeutic potential of flavonoid compounds, particularly those with specific halogen and nitro substitutions, in enhancing gut health.

## 1. Introduction

Flavonoids are a large group of compounds belonging to polyphenols, built based on a skeleton consisting of C6-C3-C6 carbon atoms, usually characterized by a cyclic system with oxygen in a three-carbon bridge [1]. They are well known for having various health-promoting properties such as antioxidant [2,3], anti-inflammatory [4,5], and antimicrobial [6,7,8,9] properties. They also have a positive effect on the human intestinal microbiome [10,11].

In recent years, the number of lifestyle diseases such as diabetes and obesity has been increasing every year. Bacterial resistance to antibiotics is also becoming a significant problem [12]. There is a pressing need to discover new compounds of natural origin and investigate their effects to combat these problems. Considerable evidence indicates that flavonoid derivatives, such as glycosides, or flavonoids with additional halogen elements or other functional groups, exhibit enhanced and/or novel health benefits. Lewin et al. [13] proved that disrupting the planarity of diosmin (which is highly insoluble and used as an oral phlebotropic drug) by adding a bromine or chlorine atom at the C-3 position significantly increases its solubility compared to the parent compound. Thebti et al. [7] proved that the structure of flavonoid compounds is essential in improving their antimicrobial activity. They showed that the presence of the -OH group at the C-2 position in chalcones, halogens (bromine, chlorine) at the C-6′ position, and the methoxy group at C-4′ have a significant impact on the reduction in the growth of Methicillin-Sensitive *Staphylococcus aureus* 60 (MSSA). Studies also report that flavonoids with nitrogen moieties may contribute to combating cancer multidrug resistance (MDR). Compounds derived from natural products are considered promising ABC transporter (ATP-binding cassette transporter) modulators in the fight against MDR. It has been shown that nitrogen-containing groups in flavonoid compounds can alter the efflux function of ABC transporters. When these transporters are overexpressed in cancer cells, they decrease the intracellular concentration of cytotoxic drugs, leading to the failure of chemotherapy treatments [14].

Furthermore, flavonoids have a great impact on the human intestinal microbiome. The human intestinal microbiome is composed of a diverse array of microorganisms that play crucial roles in digestion, immune function, and overall health. Flavonoids can modulate the composition and activity of this microbiome [11,15]. Disturbances in the composition of the human gastrointestinal microbiome are closely related to lifestyle, diet, and exposure to stress. The literature on the subject indicates significant progress in research on the composition of the intestinal microbiome and its impact on autoimmune diseases, colon cancer, dental caries, and the relationship between the microbiome and the brain (impact on depression and autism) [16,17]. Pan et al. [18] examined the influence of flavonoids on human gut microbiota in in vitro simulated fermentation. Studies showed that hesperetin-7-O-glucoside, prunin, and isoquercitrin impact the structure of human gut microbiota. Compounds could significantly enhance *Bifidobacterium* sp. (*p* < 0.05). After 24 h of in vitro simulated fermentation, the relative abundance of intestinal probiotics (e.g., *Lactobacillus* sp.) was increased by the three flavonoids and rutin. Furthermore, the relative abundance of potential pathogenic bacteria was decreased by the addition of hesperetin-7-*O*-glucoside, naringin, prunin, rutin, and isoquercitrin (e.g., *Lachnoclostridium* sp. and *Bilophila* sp.). An analysis of the impact of increased dietary flavonoid intake on the human intestinal microbiome carried out by Klinder et al. [19] demonstrated that the consumption of fruits and vegetables, along with their flavonoid and sugar content, inhibits the growth of potentially pathogenic *Clostridia* sp. The protective effects observed are likely mediated by plant-derived dietary fiber (indigestible polysaccharides) and/or flavonoids, particularly through their interactions with the gut microbiota.

Studies on the metabolism of polyphenols by the intestinal microbiota are crucial for understanding the role of these compounds and their impact on our health. Our main goal was to complement the literature about flavonoids with additional groups in specific compositions and positions in their structure. We focused on the preliminary study of biological properties, such as the influence on the growth of microorganisms and changes in the number of bacteria and yeast, in the characterized human intestinal microbiome to provide a basic screening of the selected flavonoids and allow the research community to plan future experiments. In our study, we examined the effect of chalcones, flavanones, and flavones containing -Br, -Cl, -NO_2_, and -CH_3_ on probiotic and pathogenic bacteria. We also examined the effect of the selected compounds on the standardized intestinal microbiome of healthy people and the pathologically altered intestinal microbiome. We carried out predictions using the Way2Drug PASS Online, CLC-pred, and SwissADME platforms for other possible properties of the used flavonoids.

## 2. Results

### 2.1. Microbial Research

**Figure 1:**
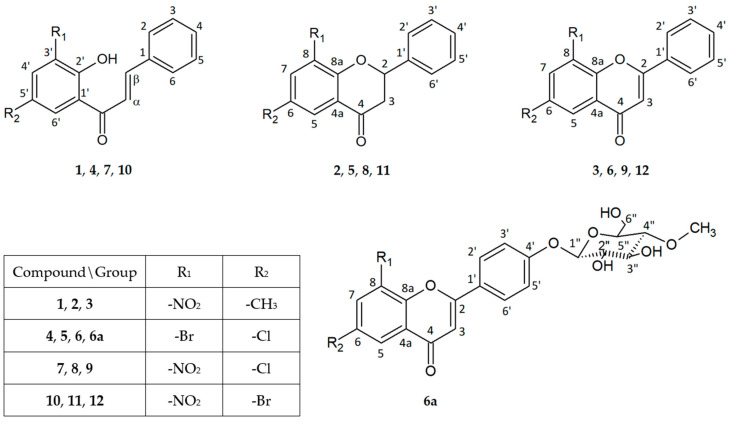
Flavonoid structures. 2′-Hydroxy-5′-methyl-3′-nitrochalcone (**1**), 6-methyl-8-nitroflavanone (**2**), 6-methyl-8-nitroflavone (**3**), 3′-bromo-5′-chloro-2′-hydroxychalcone (**4**), 8-bromo-6-chloroflavanone (**5**), 8-bromo-6-chloroflavone (**6**), 8-bromo-6-chloroflavone 4′-*O*-*β*-D-(4″-*O*-methyl)-glucopyranoside (**6a**), 5′-chloro-2′-hydroxy-3′-nitrochalcone (**7**), 6-chloro-8-nitroflavanone (**8**), 6-chloro-8-nitroflavone (**9**), 5′-bromo-2′-hydroxy-3′-nitrochalcone (**10**), 6-bromo-8-nitroflavanone (**11**), 6-bromo-8-nitroflavone (**12**).

The object of this microbiological research was flavonoids (chalcones, flavanones, and flavones) with additional -NO_2_ and -CH_3_ groups and -Br and -Cl atoms, whose structures are shown in the Figure 1. For this study, we selected twelve flavonoid aglycones (**1**)–(**12**) obtained via chemical synthesis and one glycoside (**6a**) received as a result of biotransformation in our previous studies [20,21]. We chose only (**6a**) due to the highest reaction efficiency, enabling further analyses. Detailed syntheses are described in our previous works [20,21].

**Figure 2:**
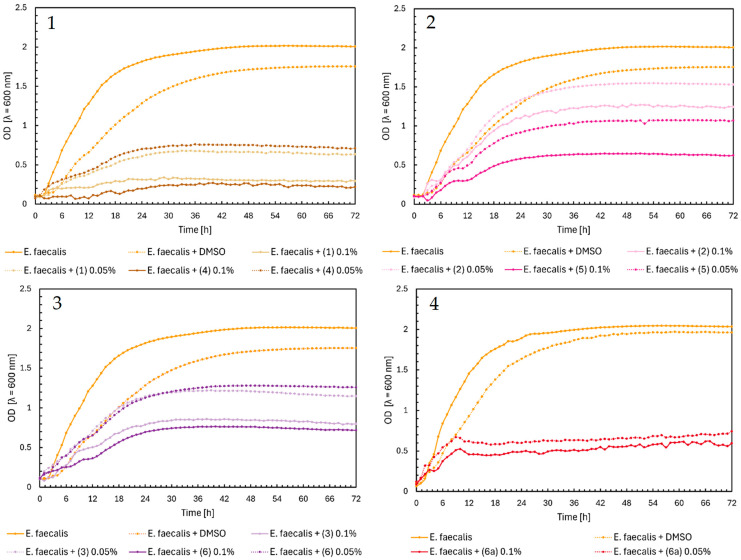
The growth kinetics of *E. faecalis* ATCC 19433 under the effects of flavonoids (**1**)–(**6**) and (**6a**). Each of Charts 1–4 contains growth of *E. faecalis* and *E. faecalis* + DMSO. Chart 1: chalcones (**1**) and (**4**) in concentrations of 0.1% and 0.05%; Chart 2: flavanones (**2**) and (**5**) in concentrations of 0.1% and 0.05%; Chart 3: flavones (**3**) and (**6**) in concentrations of 0.1% and 0.05%; Chart 4: flavone glycoside (**6a**) in concentrations of 0.1% and 0.05%.

The antimicrobial activity of flavonoids (**1**)–(**6**) and (**6a**) was studied over 72 h, with optical density readings at λ = 600 nm (OD600) taken every hour. The results are displayed in Figure 2 below. The results for the remaining compounds (**7**)–(**12**) were presented in our previous work [21].

In Figure 2, Charts 1–4, it can be noted that the highest inhibition of the growth of *Enterococcus faecalis* was shown in chalcones (**1**) and (**4**), especially in the 0.1% concentration. Furthermore, the glycoside (**6a**) showed similar properties in both concentrations (Figure 2, Chart 4). The flavanones and flavones in Figure 2, Charts 2 and 3, showed moderate inhibition of bacteria growth. The strongest was flavanone (**5**) in a concentration of 0.1%, with an average of 0.5, and flavones (**6**) and (**3**) in the same concentrations.

**Figure 3:**
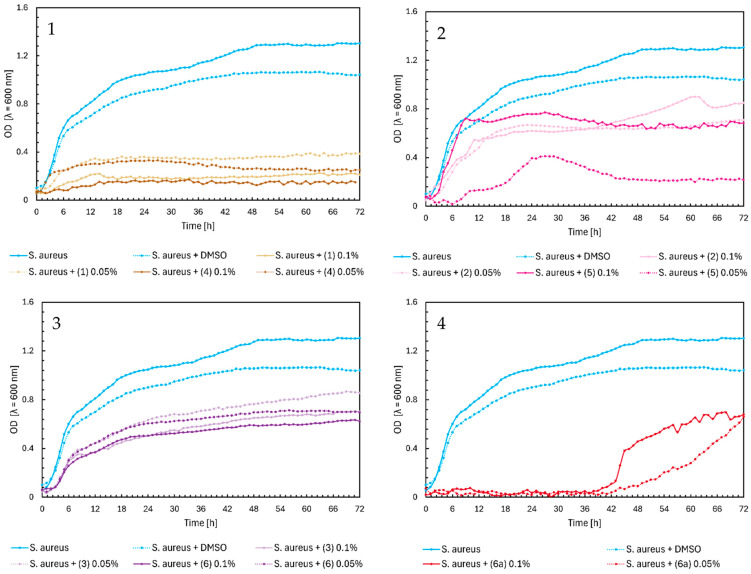
The growth kinetics of *S. aureus* ATCC 29213 under the effects of flavonoids (**1**)–(**6**) and (**6a**). Each of Charts 1–4 contains growth of *S. aureus* and *S. aureus* + DMSO. Chart 1: chalcones (**1**) and (**4**) in concentrations of 0.1% and 0.05%; Chart 2: flavanones (**2**) and (**5**) in concentrations of 0.1% and 0.05%; Chart 3: flavones (**3**) and (**6**) in concentrations of 0.1% and 0.05%; Chart 4: flavone glycoside (**6a**) in concentrations of 0.1% and 0.05%.

The maximum OD value of *Staphylococcus aureus* as a control was 1.3. In Figure 3, Chart 1, the chalcones (**1**) and (**4**) showed a high inhibitory effect on the growth of bacteria, with a maximum optical density of around 0.3. Flavanones and flavones did not significantly affect *S. aureus*. They lowered the maximum value of the optical density of bacteria to 0.8–0.7 (Figure 3, Charts 2 and 3). The highest inhibition during the first 42 h was shown by flavone glycoside (**6a**), with an optical density close to zero. After that time, there was a sudden increase in the optical density to the maximum value of around 0.6 in both concentrations. This effect may be due to bacteria carrying out various biotransformation processes or changes in the structure of the compound, which could weaken its inhibition of the growth of bacteria. However, to confirm this thesis, it would be necessary to conduct research on a larger scale and learn more about the properties of a given compound.

**Figure 4:**
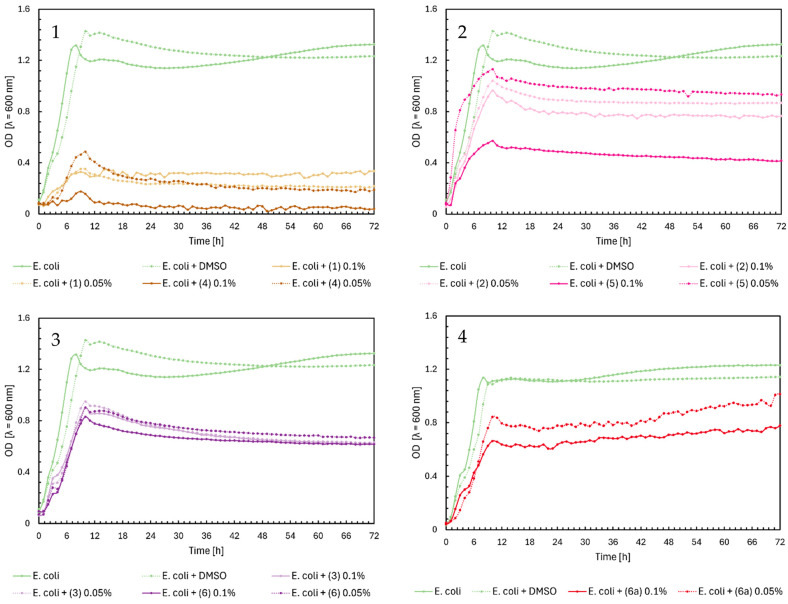
The growth kinetics of *E. coli* ATCC 25922 under the effects of flavonoids (**1**)–(**6**) and (**6a**). Each of Charts 1–4 contains growth of *E. coli* and *E. coli* + DMSO. Chart 1: chalcones (**1**) and (**4**) in concentrations of 0.1% and 0.05%; Chart 2: flavanones (**2**) and (**5**) in concentrations of 0.1% and 0.05%; Chart 3: flavones (**3**) and (**6**) in concentrations of 0.1% and 0.05%; Chart 4: flavone glycoside (**6a**) in concentrations of 0.1% and 0.05%.

In Figure 4, Charts 1–4, we observed similar growth patterns of *Escherichia coli* bacteria influenced by the tested flavonoids, consistent with the previously described results in Figure 2 and Figure 3. Chalcones (**1**) and (**4**) significantly impacted the growth of *E. coli* at both concentrations, reducing optical density (OD) from about 1.4 to 0.4. In contrast, flavanones had a much smaller inhibitory effect, with OD values remaining around 1.0, except for compound (**5**) at a 0.1% concentration, which reduced the OD to approximately 0.5 (Figure 4, Chart 2). Flavones (**3**) and (**6**), as shown in Figure 4, Chart 3, had almost identical effects at both concentrations, moderating *E. coli* growth to an OD of about 0.7–0.8, similar to glycoside (**6a**) (Figure 4, Chart 4).

**Figure 5:**
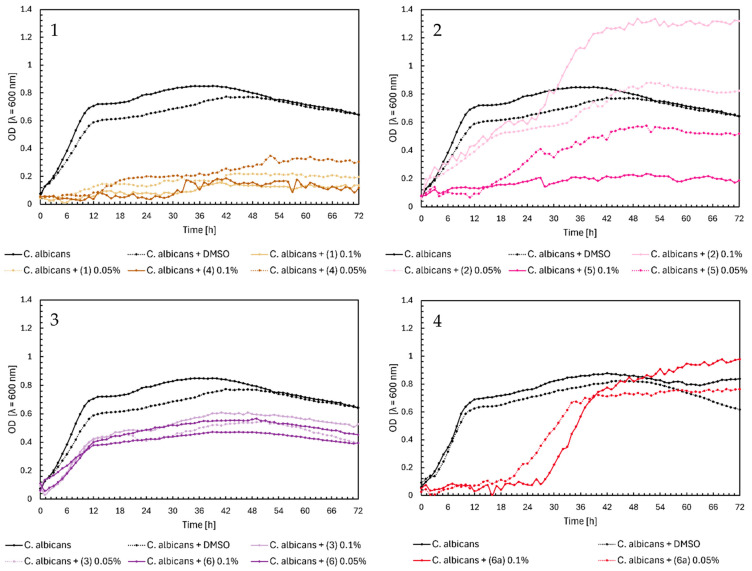
The growth kinetics of *C. albicans* ATCC 10231 under the effects of flavonoids (**1**)–(**6**) and (**6a**). Each of Charts 1–4 contains growth of *C. albicans* and *C. albicans* + DMSO. Chart 1: chalcones (**1**) and (**4**) in concentrations of 0.1% and 0.05%; Chart 2: flavanones (**2**) and (**5**) in concentrations of 0.1% and 0.05%; Chart 3: flavones (**3**) and (**6**) in concentrations of 0.1% and 0.05%; Chart 4: flavone glycoside (**6a**) in concentrations of 0.1% and 0.05%.

Noteworthy growth of *Candida albicans* was observed with flavanone (**2**) and glycoside (**6a**) in Figure 5, Charts 2 and 4. For both compounds, a sudden increase in the OD value occurred after approximately 30 h of the experiment. Analyzing the growth profile of *C. albicans* with flavanone (**2**) at 0.1%, yeast reached its maximum growth (OD = ~1.3) after two days, compared to the control’s maximum OD = 0.8. The same compound (**2**) at 0.05% did not significantly affect the yeast growth. Flavanone (**5**) at a concentration of 0.05% slightly reduced the growth of the microorganism, while at 0.1%, it completely inhibited its growth. Glycoside (**6a**) completely inhibited the growth of yeast on the first day of the experiment; after that time, the optical density increased to ~1.0 at 0.1% and ~0.8 at 0.05%. As we mentioned earlier, this may be caused by the transformation of these compounds and their modifications occurring during incubation with microorganisms. However, further research will be necessary to confirm our hypothesis. In the case of chalcones in Figure 5, Chart 1, we can observe complete inhibition of yeast growth, especially for compounds (**1**) and (**4**) at a concentration of 0.1%. Flavones (**3**) and (**6**) only weakened the growth of *C. albicans*, reaching a maximum at an optical density value of 0.4–0.6 for both concentrations (Figure 5, Chart 3).

**Figure 6:**
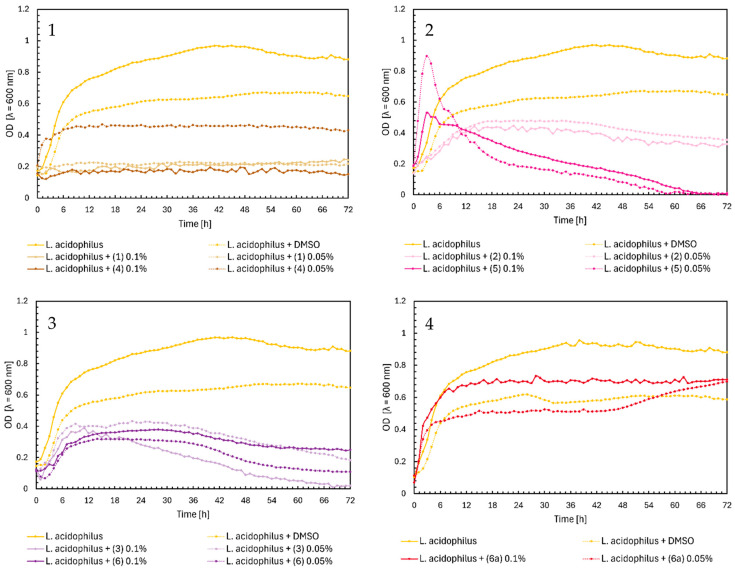
The growth kinetics of *L. acidophilus* ATCC 4356 under the effects of flavonoids (**1**)–(**6**) and (**6a**). Each of Charts 1–4 contains growth of *L. acidophilus* and *L. acidophilus* + DMSO. Chart 1: chalcones (**1**) and (**4**) in concentrations of 0.1% and 0.05%; Chart 2: flavanones (**2**) and (**5**) in concentrations of 0.1% and 0.05%; Chart 3: flavones (**3**) and (**6**) in concentrations of 0.1% and 0.05%; Chart 4: flavone glycoside (**6a**) in concentrations of 0.1% and 0.05%.

Regarding the effect of flavonoids (**1**)–(**6**) and (**6a**) on the growth of *Lactobacillus acidophilus*, chalcones and flavones significantly inhibited the bacterial growth. In Figure 6, Chart 1, all compounds except (**4**) at a concentration of 0.05%, which only reduced the bacterial growth to OD = 0.4, completely inhibited the growth. A similar result was found for flavones (**3**) and (**6**), but we observe a gradual decrease in optical density to practically zero in the final hours of the experiment (Figure 6, Chart 3). Flavanone (**2**) at both concentrations moderately inhibited the growth of *L. acidophilus*, reaching a maximum of approximately 0.5. For flavanone (**5**), in the first 3 h, there was a sharp increase in optical density and then a slow decrease to zero (Figure 6, Chart 2). Glycoside (**6a**) did not significantly affect the growth of *L. acidophilus* (Figure 6, Chart 4).

**Figure 7:**
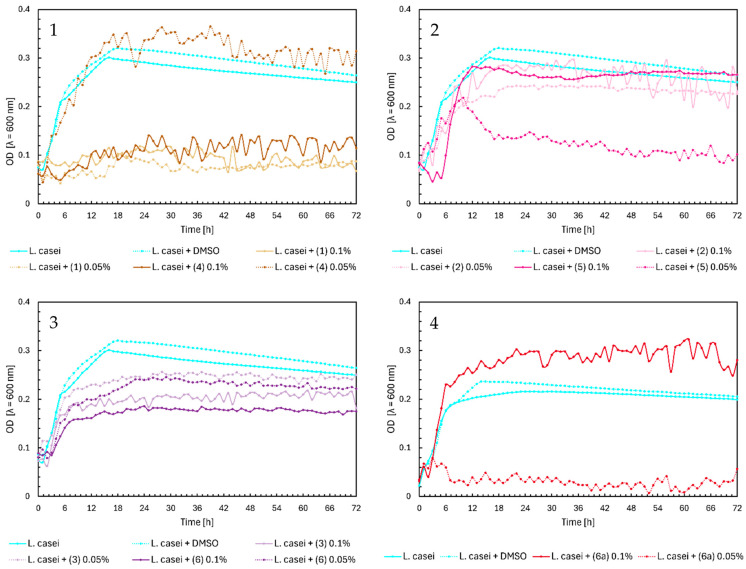
The growth kinetics of *L. casei* ATCC 393 under the effects of flavonoids (**1**)–(**6**) and (**6a**). Each of Charts 1–4 contains growth of *L. casei* and *L. casei* + DMSO. Chart 1: chalcones (**1**) and (**4**) in concentrations of 0.1% and 0.05%; Chart 2: flavanones (**2**) and (**5**) in concentrations of 0.1% and 0.05%; Chart 3: flavones (**3**) and (**6**) in concentrations of 0.1% and 0.05%; Chart 4: flavone glycoside (**6a**) in concentrations of 0.1% and 0.05%.

In Figure 7, Chart 1, chalcones (**1**) and (**4**) inhibited the growth of *Lactobacillus casei*, except for chalcone (**4**) at a 0.05% concentration, which did not significantly affect the growth of probiotic bacteria. Flavanones (**2**) and (**5**) also did not influence the optical density of *L. casei* (Figure 7, Chart 2). Flavones (**3**) and (**6**) slightly affected bacterial growth, with higher concentrations inhibiting growth more strongly, resulting in a maximum optical density of around 0.15–0.2. At a lower concentration of 0.05%, the OD was approximately 0.25 (Figure 7, Chart 3). The glycoside (**6a**) at 0.1% inducted the bacteria growth, reaching a maximum optical density at ~0.3 (Figure 7, Chart 4). 

**Figure 8:**
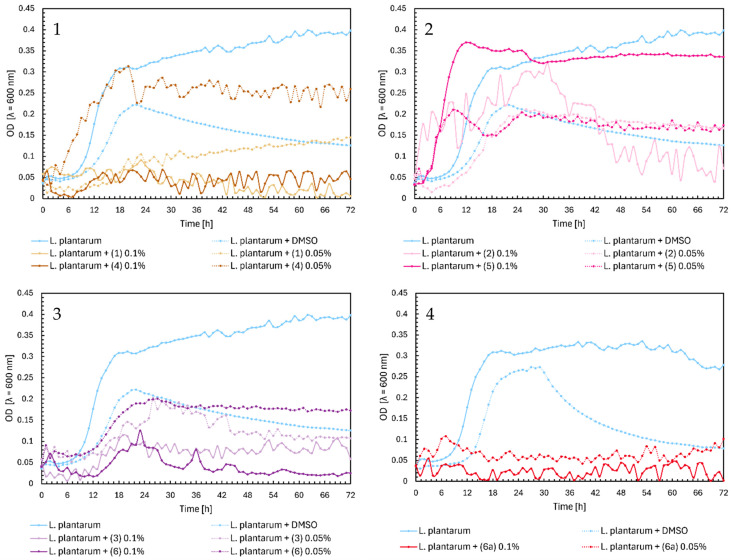
The growth kinetics of *L. plantarum* ATCC 14917 under the effects of flavonoids (**1**)–(**6**) and (**6a**). Each of Charts 1–4 contains growth of *L. plantarum* and *L. plantarum* + DMSO. Chart 1: chalcones (**1**) and (**4**) in concentrations of 0.1% and 0.05%; Chart 2: flavanones (**2**) and (**5**) in concentrations of 0.1% and 0.05%; Chart 3: flavones (**3**) and (**6**) in concentrations of 0.1% and 0.05%; Chart 4: flavone glycoside (**6a**) in concentrations of 0.1% and 0.05%.

In the case of chalcones (**1**) and (**4**), there is a similar relationship for growth as in *L. casei*. Compounds inhibited the growth of *Lactobacillus plantarum*, except for chalcone (**4**) at a 0.05% concentration, which slightly affected the growth of bacteria (Figure 8, Chart 1). In Figure 8, Chart 2, flavanone (**5**) at 0.1% did not significantly affect the growth of probiotic bacteria, which reached the maximum OD of approximately 0.37. Flavanones (**5**) at 0.05% and (**2**) at both concentrations lowered the optical density of *L. plantarum* to 0.17–0.15 at the end of the experiment, which gave a similar effect to DMSO. Flavones (**3**) and (**6**) inhibited the growth of bacteria by half (Figure 8, Chart 3), while the glycoside (**6a**) inhibited it totally (Figure 8, Chart 4).

**Figure 9:**
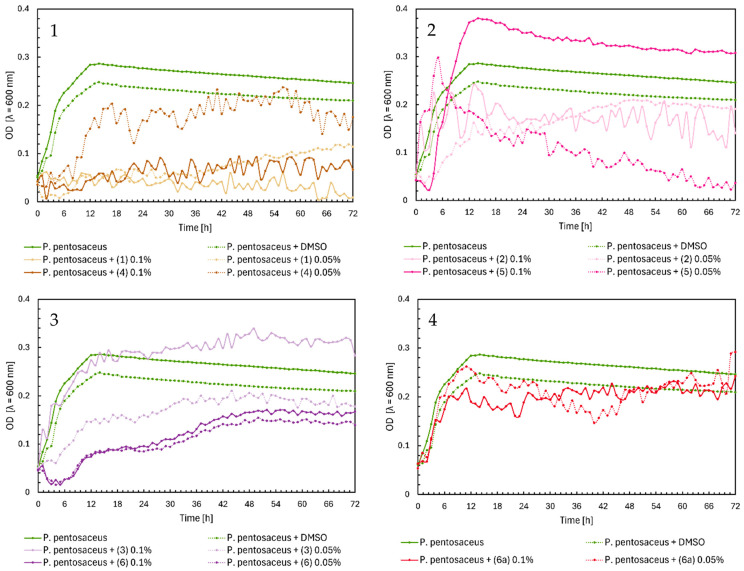
The growth kinetics of *P. pentosaceus* ATCC 33316 under the effects of flavonoids (**1**)–(**6**) and (**6a**). Each of Charts 1–4 contains growth of *P. pentosaceus* and *P. pentosaceus* + DMSO. Chart 1: chalcones (**1**) and (**4**) in concentrations of 0.1% and 0.05%; Chart 2: flavanones (**2**) and (**5**) in concentrations of 0.1% and 0.05%; Chart 3: flavones (**3**) and (**6**) in concentrations of 0.1% and 0.05%; Chart 4: flavone glycoside (**6a**) in concentrations of 0.1% and 0.05%.

Chalcones (**1**) and (**4**) inhibited the growth of *Pediococcus pentosaceus*, except for chalcone (**4**) at a 0.05% concentration (Figure 9, Chart 1). In Figure 9, Chart 2, flavanone (**5**) at 0.1% reached the highest optical density of approximately 0.4. At 0.05%, there was a sudden increase to an OD = 0.3 during the first five hours, followed by a gradual decrease to zero. Flavanone (**2**) slightly decreased bacterial growth at both concentrations. Flavone (**3**) at 0.05% slightly reduced the growth of *P. pentosaceus*, but at 0.1%, it induced bacterial growth, reaching a maximum OD = 0.33. Flavone (**6**) minimally decreased the optical density of the native microorganism at both concentrations (Figure 9, Chart 3). In Figure 9, Chart 4, glycoside (**6a**) had no effect on *P. pentosaceus*.

Summarizing our research, we found that the most active compounds in inhibiting the growth of pathogenic bacteria and yeasts were chalcones (**1**) and (**4**), particularly at concentrations of 0.05% and 0.1%. These compounds nearly completely inhibited the growth of pathogens. Glycoside (**6a**) also significantly inhibited their growth. For *S. aureus*, compound (**6a**) effectively inhibited growth until the 42nd hour of the experiment, after which there was a sudden increase in the optical density of bacteria to about 0.6 value. A similar pattern was observed for *C. albicans*, with a noticeable increase in optical density after just 24 h. Flavones (**3**) and (**6**), at both concentrations, moderately affected microorganism growth, reducing it by about half on average. Among the flavanones, compound (**5**) at a concentration of 0.1% demonstrated the best activity, significantly reducing the growth of *E. faecalis*, *E. coli*, and *C. albicans*. However, flavanone (**5**) at 0.05% and (**2**) at both concentrations showed minimal or no inhibition of pathogenic microorganism growth.

Comparing the flavonoid activity results of compounds (**1**)–(**6**) with the compounds (**7**)–(**12**) described in our previous work [21], we can emphasize that chalcones have better antimicrobial activity, followed by flavones, both aglycones, and glycoside. Flavanones exhibited the lowest activity against pathogenic microorganisms.

Concerning the impact of compounds (**1**)–(**6**) and (**6a**) on probiotic bacteria, flavone glycoside (**6a**) was able to marginally enhance the optical density of *L. casei* bacteria. It did not significantly affect the growth of *L. acidophilus* and *P. pentosaceus*, but it inhibited the growth of *L. plantarum* bacteria. Among the flavanones, compound (**5**) at 0.1% did not significantly influence the growth of probiotic bacteria, and sometimes even induced their optical density (*P. pentosaceus*). Interestingly, (**5**) at 0.1% remarkably reduced the growth of the pathogens like *E. faecalis*, *E. coli*, and *C. albicans*. The remaining flavanones (**5**) at a concentration of 0.05% and (**2**) at both concentrations did not significantly affect the growth of probiotic bacteria, limiting only the growth of *L. acidophilus*. Flavones (**3**) and (**6**) also limited the growth of *L. acidophilus* and *L. plantarum* the most, regardless of the concentration of the compounds, while they slightly limited the growth of the remaining two bacteria. The strongest inhibitory effect on probiotic bacteria had chalcones (**1**) in both concentrations and (**4**) in a concentration of 0.1%. The lower concentration of (**4**), of 0.05%, did not affect the growth of probiotic bacteria at all or slightly limited it. 

Our findings align with our previous research indicating that flavanones have the least negative impact among aglycones.

Among the tested compounds, glycoside (**6a**) at both concentrations and flavanone (**5**) at 0.1% showed the most promising results. They significantly limited or inhibited the growth of pathogenic bacteria without adversely affecting the probiotic’s growth. Notably, these compounds contained bromine and chlorine atoms. Other researchers have highlighted that the presence of halogen elements in flavonoid compounds can enhance their bactericidal properties against pathogenic bacteria [6,7,22,23].

### 2.2. Digestion In Vitro

This experiment aimed to determine the effect of flavones (**3**), (**6**), (**9**), and (**12**) on the kinetics of changes in the number of bacteria in the characterized healthy human microbiome and in the state of dysbiosis. These compounds (flavones) were selected due to the highest efficiency of the synthesis reaction and high antimicrobial activity. 

6-Methyl-8-nitroflavone (**3**) caused the most positive changes in the microbiome of people after antibiotic therapy and chemotherapy (Table 1 and Table 2). A significant increase in bacteria of the *Bifidobacterium* and *Lactobacillus* genera in the microbiome characteristic of antibiotic therapy was observed, by three orders of magnitude (5.05 ± 0.55) × 10^8^ and four orders of magnitude (1.59 ± 0.61) × 10^7^, respectively. A decrease in the count of *Clostridium* sp. by two orders of magnitude was observed compared to the control sample (normal microbiome). Furthermore, no yeast-like fungi growth was noted. In the microbiome typical for people after chemotherapy, there was an increase of three and five orders of magnitude for *Bifidobacterium* sp. (2.85 ± 1.65) × 10^7^ and *Lactobacillus* sp. (3.47 ± 3.24) × 10^8^, respectively. No growth of yeast-like fungi was observed. In the microbiome typical for people over 75 years of age, an approximate three-hundredfold increase in *Lactobacillus* sp. bacteria was observed. There was also a decrease of two orders of magnitude of *Clostridium* sp., *E. coli*, and proteolytic bacteria. Additionally, the growth of yeast-like fungi was not observed. In the microbiome of obese people, only a four-order decrease in the count of *Clostridium* sp. bacteria was observed.

Analysis of the effect of 8-bromo-6-chloroflavone (**6**), as shown in Table 3 and Table 4, showed that it caused the most significant changes in the microbiome characteristic of people after chemotherapy, increasing the number of all probiotic microorganisms: *Bifidobacterium* sp. by two orders of magnitude (3.15 ± 2.46) × 10^6^, *Lactobacillus* sp. by three orders of magnitude (4.40 ± 3.20) × 10^6^, non-pathogenic coli by two orders of magnitude (7.80 ± 2.00) × 10^5^, and *Enterococcus* sp. by two orders of magnitude (2.95 ± 0.65) × 10^4^. Regarding potentially pathogenic microorganisms, it reduced the number of proteolytic bacteria and yeast-like fungi to zero. In the microbiome typical of people after antibiotic therapy, an increase of one order of magnitude was observed for non-pathogenic *E. coli* and *Enterococcus* sp. There was also an increase in the count of *Bifidobacterium* sp. bacteria by two orders of magnitude and of *Lactobacillus* sp. by three orders of magnitude. Compound (**6**) also reduced the number of yeast-like fungi and proteolytic bacteria. In the microbiome of people over 75 years of age, there was an increase in the number of *Lactobacillus* sp. by two orders of magnitude and a significant decrease in all potentially pathogenic bacteria (*Clostridium* sp., *E. coli*, proteolytic bacteria, and yeast-like fungi). In a group of obese people, compound (**6**) reduced the number of *Clostridium* sp. bacteria by three orders of magnitude.

Upon analyzing the effect of 6-chloro-8-nitroflavone (**9**) on probiotic and potentially pathogenic microorganisms in the microbiomes of individuals over 75 years of age, significant changes were observed in the populations of specific organisms (Table 5 and Table 6). The addition of flavone (**9**) contributed to an increase in *Bifidobacterium* sp. and *Lactobacillus* sp., which comprise the human healthy intestinal microbiome, by two orders of magnitude. The compound also caused a two-order decrease in *Clostridium* sp. and *E. coli*, which are potentially pathogenic. In the microbiome of people after antibiotic therapy, the highest increase in *Bifidobacterium* sp. (by two orders of magnitude) and the complete elimination of proteolytic bacteria were observed. In the microbiome of people after chemotherapy, an increase in the number of bacteria of the *Lactobacillus* and *Enterococcus* species by two orders of magnitude was observed, as well as the elimination of proteolytic bacteria and yeast-like fungi (not observed).

In the case of 6-bromo-8-nitroflavone (**12**), it did not significantly affect the number of probiotic and potentially pathogenic microorganisms in all microbiome variants (Table 7 and Table 8). There was only a slight decrease in *Clostridium* sp. bacteria (by one order of magnitude) in the microbiome of people more than 75 y.o., and even a tenfold increase in *E. coli* bacteria in the microbiome characteristic of healthy people and after antibiotic therapy. A slight decrease of yeast-like fungi in the microbiome characteristic of people over 75 years of age was also observed.

None of the compounds (**3**), (**6**), (**9**), and (**12**) significantly influenced the composition of the probiotic and pathogenic microorganisms characteristic of healthy people (microbiome of healthy people).

### 2.3. Activity Predictions In Silico

The compounds (**1**)–(**12**) and (**6a**) underwent in silico research using platforms Way2Drug PASS Online [http://www.way2drug.com/PASSOnline, accessed on 20 May 2024] [24], CLC-Pred [https://www.way2drug.com/CLC-pred, accessed on 24 July 2024] (shown in the Supplementary Materials) and SwissADME [http://www.swissadme.ch, accessed on 20 May 2024]. Table 9 and Table 10 show probable activity and physical-chemistry analysis.

According to Pass Online analysis for potential antibacterial properties (Table 9), glycoside (**6a**) exhibits the highest probability at 53.8%, followed by chalcones (**1**), (**4**), (**7**), and (**10**), with probabilities ranging from 31% to 37%. In terms of antifungal properties, glycoside (**6a**) again shows the highest probability, reaching 66.4%. The likelihood of antifungal activity among flavonoid aglycones follows the order chalcone > flavanone > flavone. Flavonoids containing chlorine and bromine atoms (**4**), (**5**), and (**6**) have the highest Pa results. Notably, glycoside (**6a**), with a Pa value of 92%, may act as an inhibitor of the enzyme CDP-glycerol glycerophosphotransferase. This enzyme is crucial for formation of the wall teichoic acid (WTA) in Gram-positive bacteria, which are significant in pathogenesis and antibiotic resistance. Inhibiting this enzyme could prevent WTA formation, causing increased bacterial sensitivity to the substance, reducing biofilm formation, or disrupting bacterial homeostasis [25]. Simulation results support the effectiveness of glycoside (**6a**) against Gram-positive pathogenic bacteria, aligning with our experimental findings. However, this effect does not extend to probiotic bacteria, as shown in our research. When we analyze the predictions of other properties, such as anticarcinogenic, vasoprotective, or chemopreventive activity, and then consider all the compounds, the occurrence of vasoprotective properties is most likely to occur, and these are mainly flavones (**3**), (**6**), (**6a**), and (**9**). A lower probability was recorded for anticarcinogenic and chemopreventive properties, with an average result of approximately 30–25%, respectively; the exception is (**6a**), which shows 71.9% and 51.4% for chemopreventive properties.

As our studies on bacteria showed, compared to the simulation results, they do not overlap. Nevertheless, they are a good start in searching for and determining the properties of compounds that have not been known. While compound (**6a**) may, according to simulations, have good antibacterial or antifungal properties, and this is consistent with our research, we cannot say the same about compound (**5**), for which all activities had a probability of occurrence below 30%, except antifungal activity (Pa = 39.7%).

Referring to the SwissADME analyses shown in Table 10, all compounds (**1**)–(**12**) and (**6a**) have high gastrointestinal absorption and are moderately soluble in water. Additionally, they have a high bioavailability score of 0.55. This indicates that there is a 56% likelihood that each compound will achieve at least 10% oral bioavailability in rats or display measurable Caco-2 permeability [26]. None of the tested flavonoids has any contraindications for use as a drug according to the PAINS drug-likeness test, which means that the compound does not break down into fragments that may give a false positive biological result. Regarding the permeability of compounds through the blood–brain barrier (BBB permanent), flavonoids (**1**), (**6a**), (**7**), (**10**), and (**12**) do not penetrate it [27]. In silico analysis of P-glycoprotein (P-gp) substrate interactions indicates that only glycoside (**6a**) can utilize a P-glycoprotein transporter. This capability is significant for various functions, such as drug absorption, excretion, and other crucial activities that could alter the body’s response or the effects of other medications. Glycoside (**6a**) is the sole compound exhibiting this potential.

## 3. Discussion

The main objective of this research was to understand the impact of a group of flavonoid compounds (chalcones, flavanones, and flavones) with -Br, -Cl, -CH_3_, and -NO_2_ on potentially pathogenic and probiotic microbes, as well as to understand the influence on standardized normal and pathologically changed intestinal microbiome. The antibacterial and antifungal properties of flavonoids, combined with their ability to positively impact the gut microbiome, highlight their potential as therapeutic agents for maintaining homeostasis and promoting gut health.

Comparing the results of the impact on probiotic and potentially pathogenic microorganisms of the compounds presented in this study and our previous work [21], we can clearly state that the bactericidal properties of flavonoids are organized according to the following sequence: chalcones > flavones > flavanones. Such reports are also confirmed by other studies in this area [7]. This activity is correlated with the structure of chalcones—an open C ring, which has an antimicrobial effect on microorganisms [28]. Our data show that 3′-bromo-5′-chloro-2′-hydroxychalcone (**4**) had the highest antimicrobial activity and almost inhibited the growth of all tested microorganisms (*E. faecalis*, *S. aureus*, *E. coli*, *C. albicans*). Flavanones had little effect on the development of these pathogenic microorganisms, but the compound that limited their growth was 8-bromo-6-chloroflavanone (**5**). Flavones 6-methyl-8-nitroflavone (**3**) and 8-bromo-6-chloroflavone (**6**) did not show significant inhibition of pathogenic organisms, but looking at our previous results, 6-chloro-8-nitroflavone (**9**) significantly limited or inhibited their growth. 8-bromo-6-chloroflavone 4′-*O*-*β*-D-(4″-*O*-methyl)-glucopyranoside (**6a**) also showed moderate bactericidal properties. In the case of the effect of selected flavonoids and quercetin [21] on the growth of pathogenic and probiotic microorganisms, we can see that it was less bactericidal in the case of *E. faecalis* and *E. coli*. Moreover, it had a limiting effect on the growth of *L. acidophilus* and *L. casei* when compounds (**2**) and (**6a**) induced the growth (*L. casei*) or had no negative impact.

According to our results, chalcones had the strongest antimicrobial properties, and the most effective ones had bromine and chlorine atoms in their structure. Many studies also confirm that the presence of halogen atoms increases the antimicrobial and cytotoxic properties [29,30,31]. For instance, Marzec et al. [32] proved that halogenation of baicalein exhibits increased cytotoxic activity towards breast cancer cell line MCF-7 cells in comparison to natural baicalein. The presence of a nitro group also limits the growth of microorganisms [14,33]. The position of substituents in the molecule is also very crucial [28]. Studies by Wu et al. and Yi et al. showed that the presence of a lipophilic functional group, like -OCH_3_ at C-6 or C-8, increases the antibacterial properties of flavonoids. For example, tangeritin displayed more excellent activity than 5,6,7,4′-tetramethoxyflavone, with 8-OCH_3_ in the ring A being the only structural difference [34,35]. In turn, the lack of substituents in the B ring may also contribute to increased antibacterial activity. However, Avila et al. showed that, in chalcones, the methylation of the -OH group in the A ring decreased flavones’ activity or they become inactive [36]. Other studies also confirm that methylation of the -OH group in the position C-2′ and C-5′ in the A ring reduced the activity [37]. Echeverría et al. [38] highlighted that the presence of two -OH groups in the A ring and the lack of hydroxylation in the B ring contributed to strong antibacterial activity. This was demonstrated by the highly active flavanone, pinocembrin, which has stronger activity than, e.g., quercetin. The occurrence of a double bond characteristic of chalcones (α = β) and flavones between C2 = C3 increases the antibacterial activity in the Gram-positive bacteria, and the lack of this bond reduces this activity [36,38]. Our in silico studies using the PassOnline platform (and research on bacteria) also showed a high probability of antibacterial properties (53.8%) for glycoside (**6a**), which has -Br and -Cl atoms and a C2 = C3 double bond. More importantly, (**6a**) can be a CDP-glycerol glycerophosphotransferase inhibitor with a probability of 92.0%. This enzyme participates in the biosynthesis of wall teichoic acids (WTAs) in Gram-positive bacteria. WTAs are responsible for, among other things, pathogenesis and play a crucial role in bacterial resistance to antibiotics. Inhibition of CDP-glycerol glycerophosphotransferase may stop WTA formation, which may cause greater sensitivity of bacteria to antibiotics and even cell lysis [25]. SwissADME analyses indicate that only compound (**6a**) can be actively transported by P-glycoprotein (P-gp substrate). The P-gp transporter plays a key role in mediating multidrug resistance (MDR) in cancer, one of the primary mechanisms affecting chemotherapy efficacy. By utilizing the P-gp transporter, compound (**6a**) can be absorbed or perform other significant activities, potentially leading to changes in the body or altering the effects of additional medications [14,39].

Digestion in vitro research on a standardized microbiome characteristic of different groups of people showed that the most positive changes were caused by 6-methyl-8-nitroflavone (**3**) and 8-bromo-6-chloroflavone (**6**). Both compounds significantly increased the number of normal bacteria, especially in the microbiomes characteristic of people over 75 years of age, after chemotherapy and antibiotic therapy. They also reduced the number of *Clostridium* sp., proteolytic bacteria, and yeast-like fungi in these groups. Flavonoids are metabolized and/or biotransformed by the intestinal microbiota, thus producing new metabolites that promote human health by modulating the composition and structure of the intestinal flora [40]. We assume that the tested flavones (**3**), (**6**), (**9**) and (**12**), which are generally hydrophobic, may be absorbed in the stomach more effectively than flavonoid glycosides. Rechner et al. [41] showed the relationship between higher absorption of flavonoid aglycones and glycosides. During the digestion process of flavonoid compounds, various modifications may occur, e.g., decarboxylation by breaking the bond between C-2 and C-3 [42]. All kinds of biotransformation processes or modifications of flavonoids may also occur, resulting in the production of metabolites that have a beneficial effect on maintaining the proper human microbiome [43].

The precise mechanism behind the selective activity of flavonoids on pathogenic and probiotic bacteria remains undefined. Although recent years have seen a surge in research focusing on the effects of flavonoids on the intestinal microbiota, there are still several challenges in understanding the interactions between flavonoids and their impact on specific bacterial populations [40]. Flavonoids often regulate intestinal flora by promoting or inhibiting the growth of the gut microbiome. One possible explanation for this is that during flavonoid exposure to certain bacteria, particular metabolites are produced, which may be toxic to the bacteria. Flavonoids are known to enhance the growth of beneficial bacteria, potentially increasing the production of secretory immunoglobulin, which helps prevent the adhesion of pathogenic bacteria and toxins, suppresses the imbalance of other microorganisms, and maintains intestinal flora balance through competitive exclusion and the secretion of antimicrobial peptides [44].

In other studies, Liao et al. [45] explored the effects of a tea polyphenol (TP) diet on ApoE^−/−^ mice fed a high-fat diet (HFD), revealing that tea polyphenols significantly increased the population of *Bifidobacteria* in the gut. Lee et al. [46] also reported that tea polyphenols notably inhibited the growth of specific pathogenic bacteria, such as *Clostridium perfringens*, *Clostridium difficile*, and *Bacteroides* spp. In contrast, *Bifidobacterium* spp. and *Lactobacillus* spp. were comparatively less affected. From these findings, which demonstrate varying sensitivity levels among intestinal bacteria to tea polyphenols, it can be concluded that TPs promote an increase in *Bifidobacteria* numbers.

The relationship between flavonoid metabolites and the biological activity of their parent compounds remains complex and poorly understood. The precise contributions of these metabolites are still unclear.

## 4. Materials and Methods

### 4.1. Flavonoid Compunds

Flavonoid derivatives were obtained as a result of our previous research. Chalcones, flavanones, and flavones (**1**)–(**12**) were obtained thanks to the Claisen–Schmidt condensation and its further modifications, described in detail in our previous works [20,21]. 8-Bromo-6-chloroflavone 4′-*O*-*β*-D-(4″-*O*-methyl)-glucopyranoside (**6a**) was obtained in large-scale biotransformation using the *I. farinosa* KCH J2.6 strain, as also described in detail in our previous work [20].

### 4.2. Microorganisms

In the antimicrobial activity studies, the following microorganisms were used: *Enterococcus faecalis* ATCC 19433, *Staphylococcus aureus* ATCC 29213, *Escherichia coli* ATCC 25922, *Candida albicans* ATCC 10231, *Lactobacillus acidophilus* ATCC 4356, *Lactobacillus casei* ATCC 393, *Lactobacillus plantarum* ATCC 14917, *Pediococcus pentosaceus* ATCC 33316. These microorganisms were purchased from the American Type Culture Collection from the United States. Species were selected for the study encompassing both pathogenic and probiotic strains closely related with mammals. This selection aligns with the broader scope of our research team’s focus, particularly concerning the potential applications of the studied groups of compounds.

In the digestion in vitro studies, we used previously standardized intestinal microbiomes from various groups of people. The process of obtaining and determining them is presented in the following article [47]. Five standardized inoculations were used. The first group of donors were healthy people, physically active, who declared they did not take antibiotics (for a period of at least 1 year), ate rationally and followed the basic principles of healthy eating, and did not abuse alcohol (control sample); the second group of donors were people aged over 75 years of age; the third group of donors were people who completed antibiotic therapy (2–3 weeks); the fourth group of donors were people after chemotherapy (maximum one month after the end of the chemotherapy cycle); and the fifth group of donors were obese people (BMI > 30). Each of the five populations representing a specific gut microbiome was derived from donors from the groups mentioned above and contained defined groups of microorganisms: probiotic bacteria (*Lactobacillus* and *Bifidobacterium* genera), immunity-stimulating bacteria (non-pathogenic *E. coli* and *Enterococcus* genus), and potentially pathogenic microorganisms (proteolytic bacteria, the so-called putrefactive bacteria, *Clostridium* genus bacteria) and yeast-like fungi of the genus *Candida*.

### 4.3. Antimicrobial Analysis

Antimicrobial tests involved evaluating the activity of flavonoid compounds (**1**)–(**12**) and (**6a**) on the growth of selected microorganisms. For the analysis, 50 mL conical tubes were prepared with *E. faecalis* ATCC 19433, *S. aureus* ATCC 29213, *E. coli* ATCC 25922, *C. albicans* ATCC 10231, *L. acidophilus* ATCC 4356, *L. casei* ATCC 393, *L. plantarum* ATCC 14917, and *P. pentosaceus* ATCC 33316. Each tube contained 25 mL of Mueller–Hinton broth. The tubes were inoculated with approximately 1 mL of microorganisms and shaken at 120 rpm for 48 h at 37 °C, except for *C. albicans*, which was incubated at 30 °C. Flavonoid compounds (**1**)–(**12**) and (**6a**) were dissolved in DMSO at concentrations of 0.1% and 0.05%. The analysis was performed using a Synergy H1 microplate reader (BioTek Instruments, Winooski, VT, USA). Tests were conducted on a 96-well microplate with a continuous assay for 72 h, with absorbance measurements (OD600) taken every hour, including shaking breaks before each measurement (10 s, 282 cpm). Each well had a working volume of 300 µL, which included Mueller–Hinton broth, 50 µL of the microorganism, and 10 µL of the dissolved flavonoid at the specified concentration. The following controls were used: medium, medium + DMSO, medium + flavonoid dissolved in DMSO, medium + bacteria, medium + bacteria + DMSO. Research sample: medium + microorganism + flavonoid dissolved in DMSO. Data collection and analysis were performed using Imager Software Gen5 version 3.11 [21].

### 4.4. Digestion In Vitro Model

The experimental system was formed with a 1 L fermenter (Sartorius, Kostrzyn, Poland). The temperature was maintained at 37 °C. Compounds previously dissolved in DMSO at a concentration of 0.1% were added to the first stage of in vitro digestion, which aimed to simulate the conditions in the stomach (P1). For this purpose, 300 U/mL of pepsin was added to a mixture imitating gastric juice (PBS buffer with pH 7.4 (Sigma-Merck, St. Louis, MO, USA)), and the pH was lowered to 4.0 with 1 M HCl. This step was carried out for 4 h (P2). Peristaltic movements were imitated by stirring the suspension using a magnetic stirrer. The next stage was reproducing conditions in the small intestine. For this purpose, the pH of the fluid was adjusted to 6.0 using 1 M NaHCO_3_. Then, 10 mL of pancreatic-intestinal extract (P3) was added. The next step was to raise the pH to 7.4 by adding 1 M NaHCO_3_. This step was carried out for 2 h (P4). To simulate the passage of the product through the large intestine, the pH was raised to 8.0 with 2 M NaHCO_3_. Further digestion was performed under anaerobic conditions for 18 h (P5) [48]. This stage was enriched by adding an inoculation representing the intestinal microbiome mentioned in the Section 4.2. The following control was used: stomach and intestinal conditions + microbiome inoculum. Research sample: stomach and intestinal conditions + microbiome inoculum + flavonoid compound.

### 4.5. Determining the Number of Specific Microorganisms

The following microbiological media were used to determine the count of specific microorganisms in digestion in vitro studies: The total number of bacteria (5% sheep blood agar, bioMerieux, Craponne, France), number of *Bifidobacterium* sp. (agar BSM, Sigma-Merck, St. Louis, MO, USA), *Enterococcus* sp. and *E. coli* (chromogenic CPS, bioMerieux, Marcy-l’Étoile, France), potentially pathogenic *E. coli Biovare* (ENDO, Heipha, Merck, Darmstadt, Germany), anaerobic *Clostridium* sp. (TSC, Biocorp, Issoire, France), *Lactobacillus* sp. (MRS agar, Oxoid, Basingstoke, UK), *Enterobacter* spp., *Proteus* spp., *Pseudomonas* spp., *Candida* spp. (CHROMagar Candida, CHROMagar Company, Springfield, NJ, USA).

The microorganism counts were determined using the decimal dilution method (Koch method). Plates were poured with a liquefied medium dedicated to a microorganism group. After the medium solidified, the plates and samples were incubated for 48–72 h at the appropriate temperatures: 37 °C for bacteria and 30 °C for yeasts and molds. Cultures were carried out in aerobic conditions, except for bacteria of the *Bifidobacterium* genus, which were cultured in anaerobic conditions using an anaerobic chamber (Whitley MG500 Anaerobic Workstation).

### 4.6. In Silico Studies

The predictions of biological activity properties, water solubility, and drug-likeness of flavonoid derivatives based on their structural formulae were computed using Way2Drug PASS Online with accompanying services (available online: http://www.way2drug.com/PASSOnline (accessed on 20 May 2024)), CLC-Pred (available online: https://www.way2drug.com/CLC-pred (accessed on 24 July 2024)), and SwissADME (available online: http://www.swissadme.ch (accessed on 20 May 2024)). The structures of compounds were drawn with ACD/Labs Chemsketch 2018.2.1 where the SMILE format was calculated. The SMILE form was implemented in both services. The possible biological activity in PASS Online was shown as the probability to be revealed (Pa) and not to be revealed (Pi). These are independent values in the range from 0 to 1, where 0 means 0%, and 1 means 100%.

### 4.7. Statistical Analyses 

The statistical analyses were carried out for the antimicrobial analysis (unequal variances *t*-test with Holm–Bonferroni correction) and digestion in vitro studies (one-sample Student’s *t*-test with Holm–Bonferroni correction). The statistical significances, comparison of control with samples, were presented in the Supplementary Materials.

## 5. Conclusions

This research provides valuable insights into the antibacterial properties of flavonoid compounds, specifically chalcones, flavanones, and flavones, and their impact on both probiotic and pathogenic bacteria, as well as their influence on the intestinal microbiome in various health states. In the research on the activity of flavonoid compounds against probiotic and pathogenic microorganisms, chalcones demonstrated the highest antimicrobial activity among the tested flavonoid classes. The antibacterial properties of flavonoids were found to decrease in the order of chalcones > flavones > flavanones. Glycoside (**6a**) at both concentrations and flavanone (**5**) at 0.1% showed the most promising results. They significantly limited or inhibited the growth of pathogenic bacteria without adversely affecting the probiotic’s growth. The digestion in vitro model revealed that certain flavonoids, particularly 6-methyl-8-nitroflavone (**3**) and 8-bromo-6-chloroflavone (**6**), positively influenced the composition of the intestinal microbiome. These compounds significantly increased the abundance of beneficial bacteria and reduced harmful microorganisms, especially in microbiomes characteristic of elderly individuals and those recovering from chemotherapy and antibiotic therapy.

Our results highlight the significant potential of flavonoid compounds, especially those containing bromine and chlorine atoms and a nitro group, in maintaining intestinal homeostasis and promoting overall intestinal health, which may have implications for developing new antibacterial therapies and improving the effectiveness of existing treatments. This research is intended to serve as a basic screening of the selected flavonoids, complement the literature about them, and allow the research community to plan future experiments. The presented properties of flavonoids also allow a focus on several of the most active substances and the extension of biological research. Of course, further studies and knowledge of more properties of these compounds are required to determine their exact effects and use. 

## Figures and Tables

**Table 1:** The influence of 6-methyl-8-nitroflavone (**3**) on qualitative and quantitative changes in regular microorganisms of the intestinal microbiome of various origins.

Origin of the Intestinal Microbiome	The Number of Microorganisms CFU/mL									
	Control	With Compound	Control	With Compound	Control	With Compound	Control	With Compound	Control	With Compound
	Total Number of Microorganisms		*Bifidobacterium* sp.		*Lactobacillus* sp.		Nonpathogenic *E. coli*		*Enterococcus* sp.	
Microbiome of healthy people	(2.85 ± 2.36) × 10^11^	(7.75 ± 1.95) × 10^11^	(6.95 ± 0.25) × 10^9^	(3.18 ± 2.22) × 10^11^ ↑	(3.25 ± 0.95) × 10^5^	(6.75 ± 1.15) × 10^6^	(2.70 ± 0.40) × 10^6^	(8.65 ± 0.95) × 10^5^	(3.45 ± 2.25) × 10^6^	(6.40 ± 3.20) × 10^5^
Microbiome of people over 75 y.o.	(8.15 ± 3.85) × 10^9^	(4.50 ± 1.10) × 10^10^	(1.31 ± 1.19) × 10^8^	(5.55 ± 0.05) × 10^8^	(5.10 ± 1.60) × 10^4^	(9.15 ± 2.85) × 10^7^ ↑	(6.80 ± 1.00) × 10^6^	(7.35 ± 0.45) × 10^5^	(4.68 ± 3.72) × 10^5^	(9.20 ± 0.60) × 10^5^
Microbiome of people after antibiotic therapy	(3.65 ± 0.85) × 10^7^	(3.84 ± 3.06) × 10^11^ ↑	(2.30 ± 1.10) × 10^5^	(5.05 ± 0.55) × 10^8^ ↑	(2.69 ± 2.02) × 10^3^	(1.59 ± 0.61) × 10^7^ ↑	(5.95 ± 3.85) × 10^4^	(8.15 ± 1.45) × 10^5^	(4.20 ± 3.00) × 10^4^	(7.15 ± 1.55) × 10^5^
Microbiome of people after chemotherapy	(2.99 ± 2.61) × 10^7^	(3.19 ± 2.41) × 10^9^ ↑	(3.30 ± 1.20) × 10^4^	(2.85 ± 1.65) × 10^7^ ↑	(5.00 ± 2.80) × 10^3^	(3.47 ± 3.24) × 10^8^ ↑	(6.70 ± 2.20) × 10^3^	(2.40 ± 1.20) × 10^3^	(4.05 ± 0.45) × 10^2^	(5.60 ± 1.10) × 10^3^
Microbiome of obese people	(3.29 ± 2.51) × 10^8^	(3.10 ± 0.80) × 10^11^ ↑	(2.75 ± 0.65) × 10^6^	(2.37 ± 2.14) × 10^8^ ↑	(2.01 ± 1.80) × 10^7^	(4.05 ± 0.45) × 10^6^	(6.35 ± 0.95) × 10^6^	(4.20 ± 2.10) × 10^5^	(3.00 ± 1.80) × 10^5^	(7.50 ± 1.20) × 10^5^

**Table 2:** The influence of 6-methyl-8-nitroflavone (**3**) on qualitative and quantitative changes in potentially pathogenic microorganisms of the intestinal microbiome of various origins.

Origin of the Intestinal Microbiome	The Number of Microorganisms CFU/mL							
	Control	With Compound	Control	With Compound	Control	With Compound	Control	With Compound
	*Clostridium* sp.		*E. coli*		Proteolytic Bacteria		Yeast-like Fungi	
Microbiome of healthy people	(2.31 ± 2.19) × 10^3^	(3.75 ± 2.55) × 10^2^	(4.60 ± 0.30) × 10^3^	(5.05 ± 3.85) × 10^2^	(1.31 ± 1.19) × 10^3^	(1.60 ± 0.50) × 10^2^	absence	absence
Microbiome of people over 75 y.o.	(3.45 ± 1.35) × 10^5^	(2.75 ± 0.45) × 10^3^ ↓	(1.41 ± 1.39) × 10^5^	(6.45 ± 2.25) × 10^3^ ↓	(3.38 ± 3.32) × 10^4^	(1.38 ± 0.93) × 10^2^ ↓	(1.16 ± 1.04) × 10^3^	absence ↓
Microbiome of people after antibiotic therapy	(4.50 ± 2.20) × 10^5^	(2.30 ± 0.00) × 10^3^ ↓	(5.95 ± 1.85) × 10^2^	(2.30 ± 1.10) × 10^2^	(4.16 ± 4.05) × 10^3^	(1.90 ± 0.70) × 10^2^	(8.80 ± 3.20) × 10^4^	absence ↓
Microbiome of people after chemotherapy	(1.28 ± 1.03) × 10^3^	(2.30 ± 0.00) × 10^2^	(4.20 ± 1.00) × 10^2^	(3.90 ± 2.80) × 10^2^	(4.00 ± 2.80) × 10^2^	(1.70 ± 0.60) × 10^2^	(5.00 ± 0.50) × 10^2^	absence ↓
Microbiome of obese people	(2.90 ± 2.01) × 10^7^	(3.95 ± 1.65) × 10^3^ ↓	(4.52 ± 4.18) × 10^4^	(1.54 ± 0.76) × 10^3^	(3.55 ± 2.05) × 10^2^	(2.30 ± 1.10) × 10^2^	absence	absence

**Table 3:** The influence of 8-bromo-6-chloroflavone (**6**) on qualitative and quantitative changes in regular microorganisms of the intestinal microbiome of various origins.

Origin of the Intestinal Microbiome	The Number of Microorganisms CFU/mL									
	Control	With Compound	Control	With Compound	Control	With Compound	Control	With Compound	Control	With Compound
	Total Number of Microorganisms		*Bifidobacterium* sp.		*Lactobacillus* sp.		Nonpathogenic *E. coli*		*Enterococcus* sp.	
Microbiome of healthy people	(2.85 ± 2.36) × 10^11^	(4.47 ± 4.43) × 10^10^	(6.95 ± 0.25) × 10^9^	(6.70 ± 1.10) × 10^9^	(3.25 ± 0.95) × 10^5^	(3.63 ± 3.07) × 10^8^ ↑	(2.70 ± 0.40) × 10^6^	(7.25 ± 1.65) × 10^5^	(3.45 ± 2.25) × 10^6^	(4.90 ± 3.70) × 10^5^
Microbiome of people over 75 y.o.	(8.15 ± 3.85) × 10^9^	(3.74 ± 2.96) × 10^10^	(1.31 ± 1.19) × 10^8^	(7.25 ± 1.65) × 10^6^ ↓	(5.10 ± 1.60) × 10^4^	(4.50 ± 2.20) × 10^6^ ↑	(6.80 ± 1.00) × 10^6^	(5.05 ± 0.55) × 10^6^	(4.68 ± 3.72) × 10^5^	(1.15 ± 0.05) × 10^4^
Microbiome of people after antibiotic therapy	(3.65 ± 0.85) × 10^7^	(5.60 ± 1.10) × 10^8^	(2.30 ± 1.10) × 10^5^	(3.35 ± 2.25) × 10^7^ ↑	(2.69 ± 2.02) × 10^3^	(6.25 ± 0.65) × 10^6^ ↑	(5.95 ± 3.85) × 10^4^	(4.95 ± 1.75) × 10^5^	(4.20 ± 3.00) × 10^4^	(5.05 ± 3.85) × 10^5^
Microbiome of people after chemotherapy	(2.99 ± 2.61) × 10^7^	(6.70 ± 0.90) × 10^8^	(3.30 ± 1.20) × 10^4^	(3.15 ± 2.46) × 10^6^ ↑	(5.00 ± 2.80) × 10^3^	(4.40 ± 3.20) × 10^6^ ↑	(6.70 ± 2.20) × 10^3^	(7.80 ± 2.00) × 10^5^ ↑	(4.05 ± 0.45) × 10^2^	(2.95 ± 0.65) × 10^4^ ↑
Microbiome of obese people	(3.29 ± 2.51) × 10^8^	(4.84 ± 4.06) × 10^9^	(2.75 ± 0.65) × 10^6^	(7.05 ± 1.25) × 10^7^	(2.01 ± 1.80) × 10^7^	(3.95 ± 1.65) × 10^6^	(6.35 ± 0.95) × 10^6^	(1.15 ± 0.05) × 10^5^	(3.00 ± 1.80) × 10^5^	(4.70 ± 0.00) × 10^5^

**Table 4:** The influence of 8-bromo-6-chloroflavone (**6**) on qualitative and quantitative changes in potentially pathogenic microorganisms of the intestinal microbiome of various origins.

Origin of the Intestinal Microbiome	The Number of Microorganisms CFU/mL							
	Control	With Compound	Control	With Compound	Control	With Compound	Control	With Compound
	*Clostridium* sp.		*E. coli*		Proteolytic Bacteria		Yeast-like Fungi	
Microbiome of healthy people	(2.31 ± 2.19) × 10^3^	(3.40 ± 1.10) × 10^3^	(4.60 ± 0.30) × 10^3^	(7.10 ± 1.50) × 10^2^	(1.31 ± 1.19) × 10^3^	(2.30 ± 1.10) × 10^2^	absence	absence
Microbiome of people over 75 y.o.	(3.45 ± 1.35) × 10^5^	(4.40 ± 1.20) × 10^4^	(1.41 ± 1.39) × 10^5^	(2.84 ± 2.76) × 10^4^	(3.38 ± 3.32) × 10^4^	(3.57 ± 3.23) × 10^3^	(1.16 ± 1.04) × 10^3^	(5.20 ± 0.00) × 10^2^
Microbiome of people after antibiotic therapy	(4.50 ± 2.20) × 10^5^	(6.70 ± 3.10) × 10^5^	(5.95 ± 1.85) × 10^2^	(9.75 ± 5.25) × 10^2^	(4.16 ± 4.05) × 10^3^	absence ↓	(8.80 ± 3.20) × 10^4^	(5.60 ± 3.30) × 10^2^ ↓
Microbiome of people after chemotherapy	(1.28 ± 1.03) × 10^3^	(4.05 ± 0.65) × 10^4^	(4.20 ± 1.00) × 10^2^	(1.15 ± 0.05) × 10^3^	(4.00 ± 2.80) × 10^2^	absence ↓	(5.00 ± 0.50) × 10^2^	absence ↓
Microbiome of obese people	(2.90 ± 2.01) × 10^7^	(1.70 ± 0.50) × 10^4^ ↓	(4.52 ± 4.18) × 10^4^	(4.90 ± 2.70) × 10^2^ ↓	(3.55 ± 2.05) × 10^2^	(1.75 ± 0.45) × 10^2^	absence	absence

**Table 5:** The influence of 6-chloro-8-nitroflavone (**9**) on qualitative and quantitative changes in regular microorganisms of the intestinal microbiome of various origins.

Origin of the Intestinal Microbiome	The Number of Microorganisms CFU/mL									
	Control	With Compound	Control	With Compound	Control	With Compound	Control	With Compound	Control	With Compound
	Total Number of Microorganisms		*Bifidobacterium* sp.		*Lactobacillus* sp.		Nonpathogenic *E. coli*		*Enterococcus* sp.	
Microbiome of healthy people	(2.85 ± 2.36) × 10^11^	(8.70 ± 0.20) × 10^11^	(6.95 ± 0.25) × 10^9^	(5.15 ± 0.65) × 10^9^	(3.25 ± 0.95) × 10^5^	(5.95 ± 2.55) × 10^5^	(2.70 ± 0.40) × 10^6^	(3.50 ± 2.30) × 10^5^	(3.45 ± 2.25) × 10^6^	(8.15 ± 0.35) × 10^6^
Microbiome of people over 75 y.o.	(8.15 ± 3.85) × 10^9^	(5.40 ± 0.20) × 10^10^	(1.31 ± 1.19) × 10^8^	(4.60 ± 1.20) × 10^10^ ↑	(5.10 ± 1.60) × 10^4^	(7.05 ± 1.85) × 10^6^ ↑	(6.80 ± 1.00) × 10^6^	(6.50 ± 1.30) × 10^6^	(4.68 ± 3.72) × 10^5^	(3.85 ± 1.35) × 10^5^
Microbiome of people after antibiotic therapy	(3.65 ± 0.85) × 10^7^	(8.15 ± 0.35) × 10^8^	(2.30 ± 1.10) × 10^5^	(6.50 ± 2.00) × 10^7^ ↑	(2.69 ± 2.02) × 10^3^	(4.37 ± 4.14) × 10^4^	(5.95 ± 3.85) × 10^4^	(4.85 ± 3.65) × 10^5^	(4.20 ± 3.00) × 10^4^	(5.60 ± 2.20) × 10^4^
Microbiome of people after chemotherapy	(2.99 ± 2.61) × 10^7^	(5.95 ± 3.65) × 10^7^	(3.30 ± 1.20) × 10^4^	(4.50 ± 3.30) × 10^5^	(5.00 ± 2.80) × 10^3^	(4.55 ± 3.35) × 10^5^ ↑	(6.70 ± 2.20) × 10^3^	(2.25 ± 1.36) × 10^4^	(4.05 ± 0.45) × 10^2^	(6.10 ± 1.60) × 10^4^ ↑
Microbiome of obese people	(3.29 ± 2.51) × 10^8^	(5.20 ± 1.30) × 10^9^	(2.75 ± 0.65) × 10^6^	(6.05 ± 2.65) × 10^7^	(2.01 ± 1.80) × 10^7^	(4.70 ± 0.80) × 10^8^	(6.35 ± 0.95) × 10^6^	(4.30 ± 3.10) × 10^7^	(3.00 ± 1.80) × 10^5^	(5.55 ± 1.05) × 10^6^

**Table 6:** The influence of 6-chloro-8-nitroflavone (**9**) on qualitative and quantitative changes in potentially pathogenic microorganisms of the intestinal microbiome of various origins.

Origin of the Intestinal Microbiome	The Number of Microorganisms CFU/mL							
	Control	With Compound	Control	With Compound	Control	With Compound	Control	With Compound
	*Clostridium* sp.		*E. coli*		Proteolytic Bacteria		Yeast-like Fungi	
Microbiome of healthy people	(2.31 ± 2.19) × 10^3^	(4.60 ± 2.10) × 10^2^	(4.60 ± 0.30) × 10^3^	(4.58 ± 4.33) × 10^4^	(1.31 ± 1.19) × 10^3^	(1.16 ± 0.64) × 10^3^	absence	absence
Microbiome of people over 75 y.o.	(3.45 ± 1.35) × 10^5^	(7.35 ± 1.55) × 10^3^ ↓	(1.41 ± 1.39) × 10^5^	(3.20 ± 2.00) × 10^3^ ↓	(3.38 ± 3.32) × 10^4^	(4.05 ± 1.55) × 10^3^	(1.16 ± 1.04) × 10^3^	(4.55 ± 0.65) × 10^2^
Microbiome of people after antibiotic therapy	(4.50 ± 2.20) × 10^5^	(5.25 ± 4.36) × 10^5^	(5.95 ± 1.85) × 10^2^	(6.05 ± 2.85) × 10^2^	(4.16 ± 4.05) × 10^3^	absence ↓	(8.80 ± 3.20) × 10^4^	(3.07 ± 2.73) × 10^4^
Microbiome of people after chemotherapy	(1.28 ± 1.03) × 10^3^	(4.02 ± 3.79) × 10^3^	(4.20 ± 1.00) × 10^2^	(1.90 ± 0.40) × 10^2^	(4.00 ± 2.80) × 10^2^	absence ↓	(5.00 ± 0.50) × 10^2^	absence ↓
Microbiome of obese people	(2.90 ± 2.01) × 10^7^	(6.05 ± 2.65) × 10^6^	(4.52 ± 4.18) × 10^4^	(5.05 ± 2.75) × 10^3^	(3.55 ± 2.05) × 10^2^	(2.56 ± 2.35) × 10^3^	absence	absence

**Table 7:** The influence of 6-bromo-8-nitroflavone (**12**) on qualitative and quantitative changes in regular microorganisms of the intestinal microbiome of various origins.

Origin of the Intestinal Microbiome	The Number of Microorganisms CFU/mL									
	Control	With Compound	Control	With Compound	Control	With Compound	Control	With Compound	Control	With Compound
	Total Number of Microorganisms		*Bifidobacterium* sp.		*Lactobacillus* sp.		Nonpathogenic *E. coli*		*Enterococcus* sp.	
Microbiome of healthy people	(2.85 ± 2.36) × 10^11^	(5.20 ± 3.70) × 10^11^	(6.95 ± 0.25) × 10^9^	(2.90 ± 1.60) × 10^9^	(3.25 ± 0.95) × 10^5^	(5.90 ± 3.00) × 10^5^	(2.70 ± 0.40) × 10^6^	(2.05 ± 0.25) × 10^6^	(3.45 ± 2.25) × 10^6^	(1.16 ± 1.04) × 10^6^
Microbiome of people over 75 y.o.	(8.15 ± 3.85) × 10^9^	(5.05 ± 0.55) × 10^11^ ↑	(1.31 ± 1.19) × 10^8^	(4.95 ± 2.85) × 10^8^	(5.10 ± 1.60) × 10^4^	(3.90 ± 0.60) × 10^4^	(6.80 ± 1.00) × 10^6^	(3.20 ± 2.00) × 10^6^	(4.68 ± 3.72) × 10^5^	(6.65 ± 1.15) × 10^5^
Microbiome of people after antibiotic therapy	(3.65 ± 0.85) × 10^7^	(3.60 ± 1.10) × 10^8^	(2.30 ± 1.10) × 10^5^	(3.50 ± 2.30) × 10^5^	(2.69 ± 2.02) × 10^3^	(5.85 ± 0.05) × 10^3^	(5.95 ± 3.85) × 10^4^	(3.53 ± 3.17) × 10^5^	(4.20 ± 3.00) × 10^4^	(5.90 ± 3.00) × 10^4^
Microbiome of people after chemotherapy	(2.99 ± 2.61) × 10^7^	(3.35 ± 2.46) × 10^6^	(3.30 ± 1.20) × 10^4^	(4.42 ± 4.19) × 10^4^	(5.00 ± 2.80) × 10^3^	(6.60 ± 1.20) × 10^3^	(6.70 ± 2.20) × 10^3^	(2.85 ± 1.96) × 10^3^	(4.05 ± 0.45) × 10^2^	(4.05 ± 2.85) × 10^2^
Microbiome of obese people	(3.29 ± 2.51) × 10^8^	(5.15 ± 0.65) × 10^8^	(2.75 ± 0.65) × 10^6^	(3.87 ± 3.53) × 10^6^	(2.01 ± 1.80) × 10^7^	(3.96 ± 3.84) × 10^7^	(6.35 ± 0.95) × 10^6^	(1.83 ± 1.38) × 10^6^	(3.00 ± 1.80) × 10^5^	(7.15 ± 1.55) × 10^5^

**Table 8:** The influence of 6-bromo-8-nitroflavone (**12**) on qualitative and quantitative changes in potentially pathogenic microorganisms of the intestinal microbiome of various origins.

Origin of the Intestinal Microbiome	The Number of Microorganisms CFU/mL							
	Control	With Compound	Control	With Compound	Control	With Compound	Control	With Compound
	*Clostridium* sp.		*E. coli*		Proteolytic Bacteria		Yeast-like Fungi	
Microbiome of healthy people	(2.31 ± 2.19) × 10^3^	(1.25 ± 0.05) × 10^2^	(4.60 ± 0.30) × 10^3^	(2.25 ± 1.15) × 10^4^	(1.31 ± 1.19) × 10^3^	(6.20 ± 0.30) × 10^3^	absence	absence
Microbiome of people over 75 y.o.	(3.45 ± 1.35) × 10^5^	(7.70 ± 1.90) × 10^4^	(1.41 ± 1.39) × 10^5^	(3.50 ± 2.30) × 10^5^	(3.38 ± 3.32) × 10^4^	(7.25 ± 1.65) × 10^4^	(1.16 ± 1.04) × 10^3^	(7.70 ± 4.30) × 10^2^
Microbiome of people after antibiotic therapy	(4.50 ± 2.20) × 10^5^	(2.35 ± 0.85) × 10^5^	(5.95 ± 1.85) × 10^2^	(4.05 ± 3.76) × 10^3^	(4.16 ± 4.05) × 10^3^	(3.50 ± 2.30) × 10^3^	(8.80 ± 3.20) × 10^4^	(1.29 ± 0.51) × 10^4^
Microbiome of people after chemotherapy	(1.28 ± 1.03) × 10^3^	(3.55 ± 1.25) × 10^2^	(4.20 ± 1.00) × 10^2^	(3.00 ± 1.50) × 10^2^	(4.00 ± 2.80) × 10^2^	(7.80 ± 2.00) × 10^2^	(5.00 ± 0.50) × 10^2^	(4.70 ± 3.10) × 10^2^
Microbiome of obese people	(2.90 ± 2.01) × 10^7^	(3.14 ± 2.36) × 10^7^	(4.52 ± 4.18) × 10^4^	(5.25 ± 4.36) × 10^4^	(3.55 ± 2.05) × 10^2^	(2.75 ± 0.65) × 10^2^	absence	absence

**Table 9:** PASS Online predictions. Pa—probable activity; Pi—probable inactivity. Values range from 0 to 1, where 1 represents 100% probability of Pa or Pi, and 0 represents 0% probability of Pa or Pi. The value x means that no information was available in the system.

Compound	Probability	Antibacterial	Anticarcinogenic	Vasoprotector	Chemopreventive	CDP-glycerol Glycerophosphotransferase Inhibitor	Bacterial Efflux Pomp Inhibitor	Antifungal
(**1**)	Pa	0.373	0.309	0.491	0.316	0.336	x	0.453
	Pi	0.037	0.055	0.042	0.034	0.264	x	0.039
(**2**)	Pa	0.216	0.322	0.372	0.280	x	x	0.346
	Pi	0.104	0.050	0.093	0.040	x	x	0.064
(**3**)	Pa	0.212	0.302	0.707	0.239	x	x	0.280
	Pi	0.107	0.057	0.010	0.052	x	x	0.090
(**4**)	Pa	0.312	0.210	0.340	0.212	x	0.124	0.539
	Pi	0.056	0.109	0.116	0.062	x	0.082	0.025
(**5**)	Pa	0.212	0.233	0.256	0.177	x	0.133	0.397
	Pi	0.107	0.092	0.232	0.076	x	0.050	0.050
(**6**)	Pa	0.209	0.216	0.497	0.146	x	0.133	0.324
	Pi	0.109	0.104	0.041	0.096	x	0.049	0.072
(**6a**)	Pa	0.538	0.719	0.828	0.514	0.920	x	0.664
	Pi	0.013	0.008	0.004	0.014	0.007	x	0.012
(**7**)	Pa	0.332	0.247	0.355	0.242	x	0.127	0.499
	Pi	0.049	0.084	0.104	0.051	x	0.071	0.031
(**8**)	Pa	0.160	0.247	0.265	0.208	x	0.135	0.384
	Pi	0.155	0.084	0.214	0.063	x	0.043	0.053
(**9**)	Pa	x	0.228	0.516	0.169	x	0.136	0.313
	Pi	x	0.096	0.035	0.080	x	0.042	0.076
(**10**)	Pa	0.365	0.207	0.268	0.188	x	x	0.464
	Pi	0.039	0.112	0.208	0.070	x	x	0.037
(**11**)	Pa	0.207	0.216	x	0.169	x	x	0.325
	Pi	0.111	0.104	x	0.081	x	x	0.072
(**12**)	Pa	0.203	0.202	0.381	0.139	x	x	0.263
	Pi	0.114	0.118	0.087	0.102	x	x	0.099

**Table 10:** SwissADME predictions. GI absorption—gastrointestinal absorption; BBB permeant—blood-brain barrier permeant; P-gp substrate—P-glycoprotein substrate; PAINS—pan assay interference, for compounds identification of potentially problematic fragments (also known as frequent hitters or promiscuous compounds).

Compound	GI Absorption	BBB Permeant	P-gp Substrate	Water Solubility (Ali)	Bioavailability Score	PAINS
(**1**)	High	No	No	Moderately soluble	0.55	0 alert
(**2**)	High	Yes	No	Moderately soluble	0.55	0 alert
(**3**)	High	Yes	No	Moderately soluble	0.55	0 alert
(**4**)	High	Yes	No	Moderately soluble	0.55	0 alert
(**5**)	High	Yes	No	Moderately soluble	0.55	0 alert
(**6**)	High	Yes	No	Moderately soluble	0.55	0 alert
(**6a**)	High	No	Yes	Moderately soluble	0.55	0 alert
(**7**)	High	No	No	Moderately soluble	0.55	0 alert
(**8**)	High	Yes	No	Moderately soluble	0.55	0 alert
(**9**)	High	Yes	No	Moderately soluble	0.55	0 alert
(**10**)	High	No	No	Moderately soluble	0.55	0 alert
(**11**)	High	Yes	No	Moderately soluble	0.55	0 alert
(**12**)	High	No	No	Moderately soluble	0.55	0 alert

## Data Availability

Samples of the compounds (**1**)–(**12**), and (**6a**) are available from the authors.

## Supplementary Materials

The following supporting information can be downloaded at: https://www.mdpi.com/article/10.3390/ijms25179269/s1.
