# Supplementary material for: The Influence of Flavonoids with -Br, -Cl Atoms and -NO2, -CH3 Groups on the Growth Kinetics and the Number of Pathogenic and Probiotic Microorganisms
Source: Int J Mol Sci. 2024 Aug 27;25(17):9269. doi: 10.3390/ijms25179269 (PMC11395712; doi:10.3390/ijms25179269)
Supplement: Supplementary file 1 [file ijms-25-09269-s001.zip › ijms-3118281-supplementary.pdf]

Supplementary materials

The Influence of Flavonoids with -Br, -Cl Atoms and -NO_2, -CH_3 Groups on The Growth Kinetics and The Number of Pathogenic and Probiotic Microorganisms

Martyna Perz [1,*], Daria Szymanowska [2,3] and Edyta Kostrzewa-Susłow [1,*]

[1] Department of Food Chemistry and Biocatalysis, Faculty of Biotechnology and Food Science, Wrocław University of Environmental and Life Sciences, 50-375 Wrocław, Poland
[2] Department of Biotechnology and Food Microbiology, Faculty of Food Science and Nutrition, Poznań University of Life Sciences, 60-627 Poznań, Poland; daria.szymanowska@up.poznan.pl
[3] Department of Pharmacognosy and Biomaterials, Faculty of Pharmacy, Poznań University of Medical Sciences, 60-806 Poznań, Poland; dszymanowska@ump.edu.pl
* Correspondence: martyna.perz@upwr.edu.pl (M.P.); edyta.kostrzewa-suslow@upwr.edu.pl (E.K.-S.)

Table of contents

Figure S 1. 2′-Hydroxy-5′-methyl-3′-nitrochalcone (**1**) activity prediction using SwissADME. 2
Figure S 2. 6-Methyl-8-nitroflavanone (**2**) activity prediction using SwissADME. 3
Figure S 3. 6-Methyl-8-nitroflavone (**3**) activity prediction using SwissADME. 4
Figure S 4. 3′-Bromo-5′-chloro-2′-hydroxychalcone (**4**) activity prediction using SwissADME. 5
Figure S 5. 8-Bromo-6-chloroflavanone (**5**) activity prediction using SwissADME. 6
Figure S 6. 8-Bromo-6-chloroflavone (**6**) activity prediction using SwissADME. 7
Figure S 7. 8-Bromo-6-chloroflavone 4′-O-β-D-(4″-O-methyl)-glucopyranoside (**6a**) activity prediction using SwissADME. 8
Figure S 8. 5′-Chloro-2′-hydroxy-3′-nitrochalcone (**7**) activity prediction using SwissADME. 9
Figure S 9. 6-Chloro-8-nitroflavanone (**8**) activity prediction using SwissADME. 10
Figure S 10. 6-Chloro-8-nitroflavone (**9**) activity prediction using SwissADME. 11
Figure S 11. 5′-Bromo-2′-hydroxy-3′-nitrochalcone (**10**) activity prediction using SwissADME. 12
Figure S 12. 6-Bromo-8-nitroflavanone (**11**) activity prediction using SwissADME. 13
Figure S 13. 6-Bromo-8-nitroflavone (**12**) activity prediction using SwissADME. 14

Table S 1. 2′-Hydroxy-5′-methyl-3′-nitrochalcone (**1**) cytotoxicity prediction using Way2Drug CLC-pred. 2
Table S 2. 6-Methyl-8-nitroflavanone (**2**) cytotoxicity prediction using Way2Drug CLC-pred. 3
Table S 3. 6-Methyl-8-nitroflavone (**3**) cytotoxicity prediction using Way2Drug CLC-pred. 4
Table S 4. 3′-Bromo-5′-chloro-2′-hydroxychalcone (**4**) cytotoxicity prediction using Way2Drug CLC-pred. 5
Table S 5. 8-Bromo-6-chloroflavanone (**5**) cytotoxicity prediction using Way2Drug CLC-pred. 6
Table S 6. 8-Bromo-6-chloroflavone (**6**) cytotoxicity prediction using Way2Drug CLC-pred. 7
Table S 7. 8-Bromo-6-chloroflavone 4′-O-β-D-(4″-O-methyl)-glucopyranoside (**6a**) cytotoxicity prediction using Way2Drug CLC-pred. 8
Table S 8. 5′-Chloro-2′-hydroxy-3′-nitrochalcone (**7**) cytotoxicity prediction using Way2Drug CLC-pred. 9
Table S 9. 6-Chloro-8-nitroflavanone (**8**) cytotoxicity prediction using Way2Drug CLC-pred. 10
Table S 10. 6-Chloro-8-nitroflavone (**9**) cytotoxicity prediction using Way2Drug CLC-pred. 11
Table S 11. 5′-Bromo-2′-hydroxy-3′-nitrochalcone (**10**) cytotoxicity prediction using Way2Drug CLC-pred. 12
Table S 12. 6-Bromo-8-nitroflavanone (**11**) cytotoxicity prediction using Way2Drug CLC-pred. 13
Table S 13. 6-Bromo-8-nitroflavone (**12**) cytotoxicity prediction using Way2Drug CLC-pred. 14
Table S 14. Unequal variances t-test with Holm–Bonferroni correction for E. faecalis. 15
Table S 15. Unequal variances t-test with Holm–Bonferroni correction for S. aureus. 15
Table S 16. Unequal variances t-test with Holm–Bonferroni correction for E. coli. 15
Table S 17. Unequal variances t-test with Holm–Bonferroni correction for C. albicans. 16
Table S 18. Unequal variances t-test with Holm–Bonferroni correction for L. acidophilus. 16
Table S 19. Unequal variances t-test with Holm–Bonferroni correction for L. casei. 16
Table S 20. Unequal variances t-test with Holm–Bonferroni correction for L. plantarum. 17
Table S 21. Unequal variances t-test with Holm–Bonferroni correction for P. pentosaceus. 17
Table S 22. One-sample Student's t-test with Holm–Bonferroni correction for digestion in vitro studies. 18

Compound name: 2′-Hydroxy-5′-methyl-3′-nitrochalcone
Symbol number: **1**
Molecular Formula: $C_{16}H_{13}NO_4$
Formula Weight: 283.279
SMILE: O=[N+]([O-])c1cc(C)cc(C(=O)/C=C/c2ccccc2)c1O

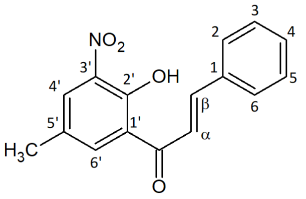

*Table S 1. 2′-Hydroxy-5′-methyl-3′-nitrochalcone (**1**) cytotoxicity prediction using Way2Drug CLC-pred.*

Lp.	Pa	Pi	Cell-line	Description	Tissue/Organ	Type
1	0.722	0.060	A2780cisR	Cisplatin-resistant ovarian carcinoma	Ovarium	Carcinoma
2	0.545	0.020	K562	Erythroleukemia	Haematopoietic and lymphoid tissue	Leukemia
3	0.507	0.007	RCC4	Clear cell renal cell carcinoma	Kidney	Carcinoma
4	0.471	0.024	SNU-475	Hepatocellular carcinoma	Liver	Carcinoma
5	0.425	0.055	YAPC	Pancreatic carcinoma	Pancreas	Carcinoma
6	0.393	0.033	HEK293	Embryonic kidney fibroblast	Kidney	Normal
7	0.388	0.054	CAL-51	Breast carcinoma	Breast	Carcinoma
8	0.373	0.123	NCI-H441	Papillary adenocarcinoma	Lung	Adenocarcinoma
9	0.351	0.169	SW48	Colorectal Adenocarcinoma	Colon	Adenocarcinoma
10	0.346	0.023	RL	Non-Hodgkin's Lymphoma	Ascites	Lymphoma

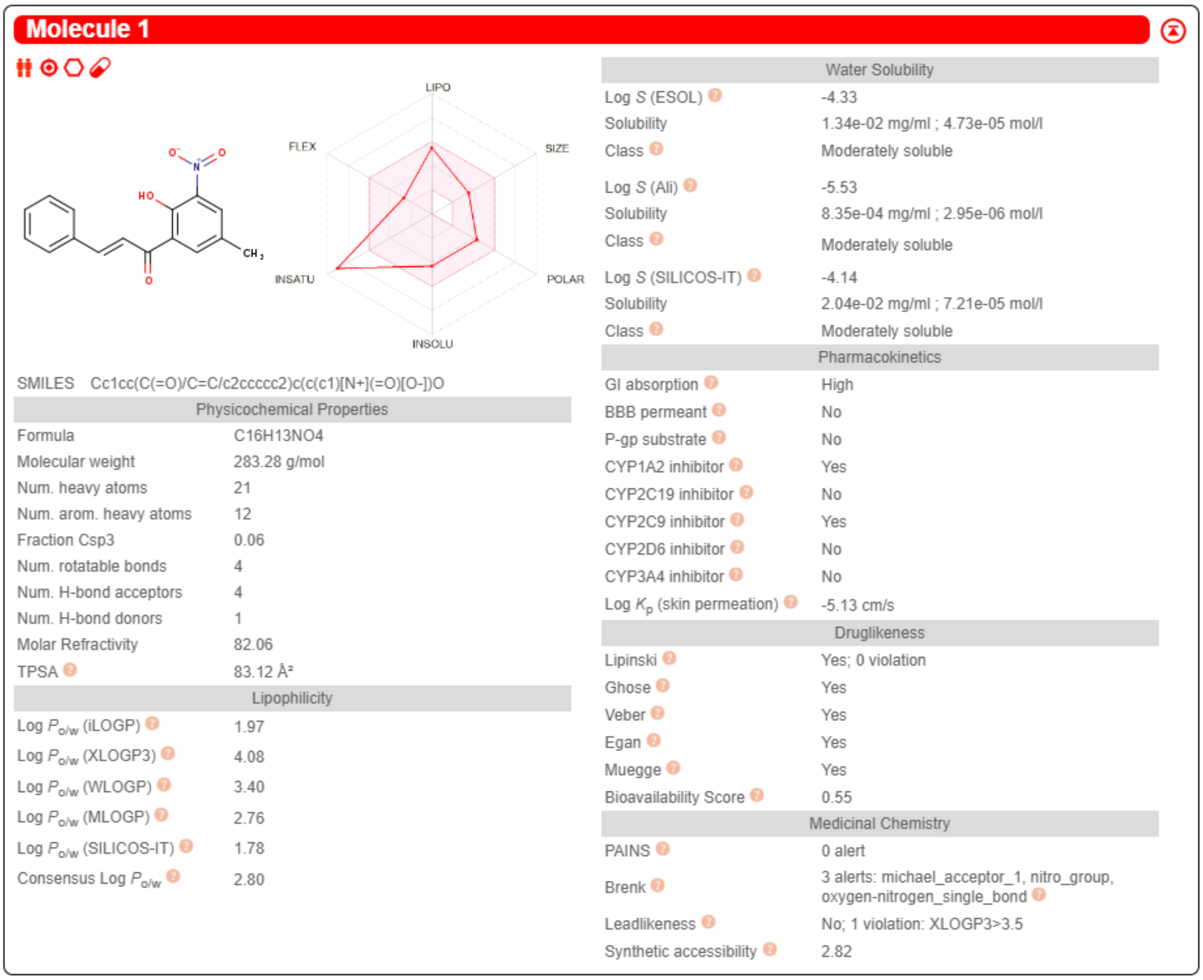
Molecule 1

SMILES Cc1cc(C(=O)/C=C/c2ccccc2)c(c(c1)[N+](=O)[O-])O

Physicochemical Properties	
Formula	C16H13NO4
Molecular weight	283.28 g/mol
Num. heavy atoms	21
Num. arom. heavy atoms	12
Fraction Csp3	0.06
Num. rotatable bonds	4
Num. H-bond acceptors	4
Num. H-bond donors	1
Molar Refractivity	82.06
TPSA	83.12 Å²
**Lipophilicity**	
Log $P_{o/w}$ (iLOGP)	1.97
Log $P_{o/w}$ (XLOGP3)	4.08
Log $P_{o/w}$ (WLOGP)	3.40
Log $P_{o/w}$ (MLOGP)	2.76
Log $P_{o/w}$ (SILICOS-IT)	1.78
Consensus Log $P_{o/w}$	2.80

Water Solubility	
Log *S* (ESOL)	-4.33
Solubility	1.34e-02 mg/ml ; 4.73e-05 mol/l
Class	Moderately soluble
Log *S* (Ali)	-5.53
Solubility	8.35e-04 mg/ml ; 2.95e-06 mol/l
Class	Moderately soluble
Log *S* (SILICOS-IT)	-4.14
Solubility	2.04e-02 mg/ml ; 7.21e-05 mol/l
Class	Moderately soluble
**Pharmacokinetics**	
GI absorption	High
BBB permeant	No
P-gp substrate	No
CYP1A2 inhibitor	Yes
CYP2C19 inhibitor	No
CYP2C9 inhibitor	Yes
CYP2D6 inhibitor	No
CYP3A4 inhibitor	No
Log K_p (skin permeation)	-5.13 cm/s
**Druglikeness**	
Lipinski	Yes; 0 violation
Ghose	Yes
Veber	Yes
Egan	Yes
Muegge	Yes
Bioavailability Score	0.55
**Medicinal Chemistry**	
PAINS	0 alert
Brenk	3 alerts: michael_acceptor_1, nitro_group, oxygen-nitrogen_single_bond
Leadlikeness	No; 1 violation: XLOGP3>3.5
Synthetic accessibility	2.82

*Figure S 1. 2′-Hydroxy-5′-methyl-3′-nitrochalcone (**1**) activity prediction using SwissADME.*

Compound name: 6-Methyl-8-nitroflavanone
Symbol: **2**
Molecular Formula: $C_{16}H_{13}NO_4$
Formula Weight: 283.279
SMILE: [O-][N+](=O)c1cc(C)cc2c1OC(CC2=O)c1ccccc1

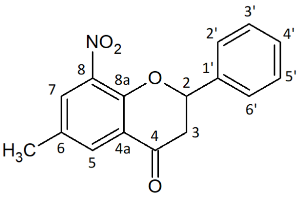

*Table S 2. 6-Methyl-8-nitroflavanone (**2**) cytotoxicity prediction using Way2Drug CLC-pred.*

Lp.	Pa	Pi	Cell-line	Description	Tissue/Organ	Type
1	0.759	0.046	A2780cisR	Cisplatin-resistant ovarian carcinoma	Ovarium	Carcinoma
2	0.596	0.004	MES-SA	Uterine corpus sarcoma	Uterus	Sarcoma
3	0.396	0.087	YAPC	Pancreatic carcinoma	Pancreas	Carcinoma
4	0.391	0.044	NCI-H187	Small cell lung carcinoma	Lung	Carcinoma
5	0.346	0.031	5637	Urothelial bladder carcinoma	Urinary tract	Carcinoma
6	0.324	0.036	NALM-6	Adult B acute lymphoblastic leukemia	Haematopoietic and lymphoid tissue	Leukemia
7	0.321	0.150	SK-MES-1	Squamous cell lung carcinoma	Lung	Carcinoma
8	0.313	0.114	HCC1806	Acantholytic Squamous Cell Carcinoma	Breast	Carcinoma
9	0.305	0.037	C8166	Leukemic T-cells	Blood	Leukemia
10	0.304	0.187	SNU-475	Hepatocellular carcinoma	Liver	Carcinoma

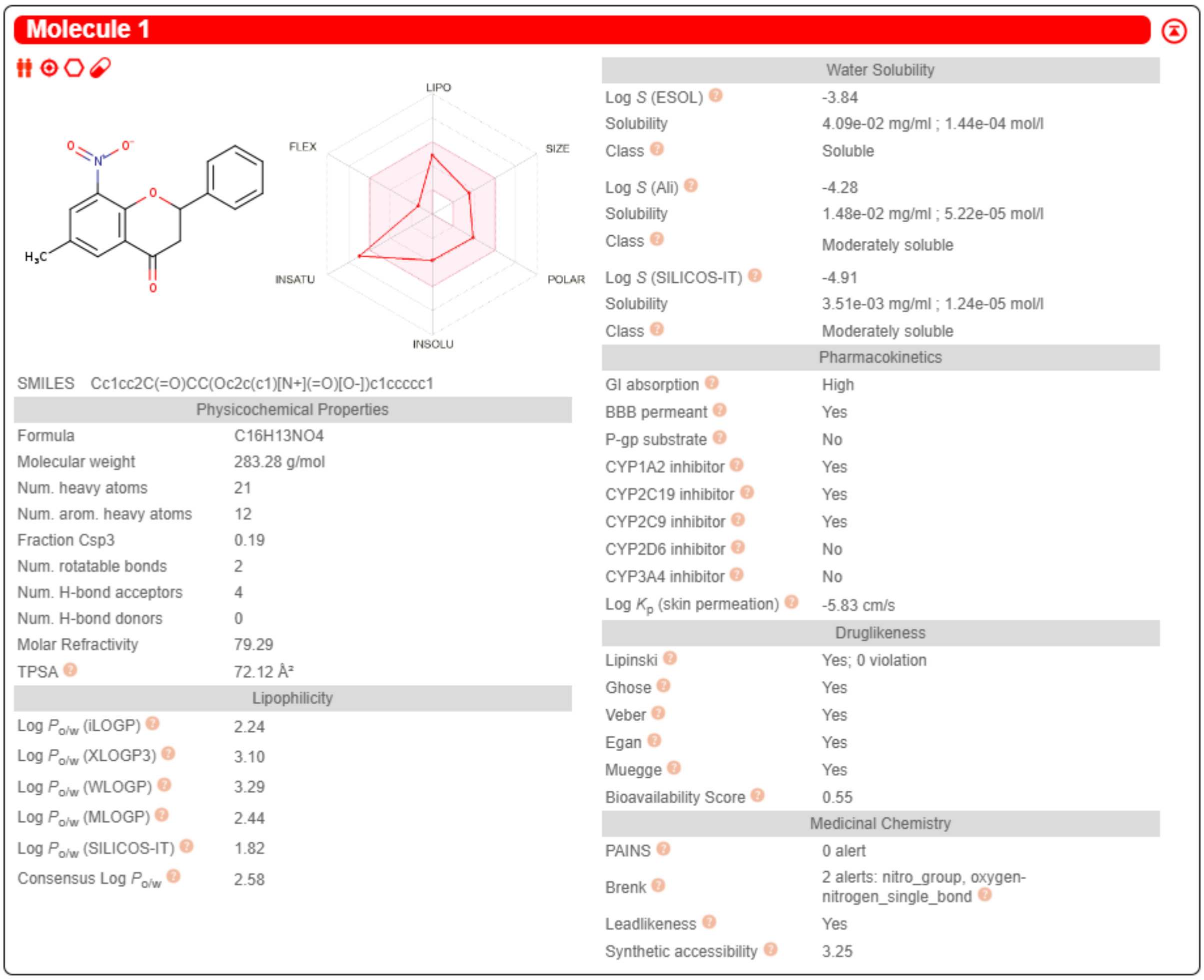

*Figure S 2. 6-Methyl-8-nitroflavanone (**2**) activity prediction using SwissADME.*

Compound name: 6-Methyl-8-nitroflavone

Symbol: **3**

Molecular Formula: $C_{16}H_{11}NO_4$

Formula Weight: 281.263

SMILE: [O-][N+](=O)c1cc(C)cc2c1OC(=CC2=O)c1ccccc1

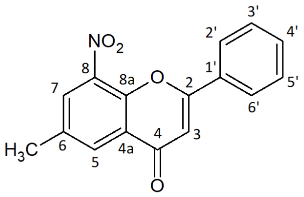

*Table S 3. 6-Methyl-8-nitroflavone (**3**) cytotoxicity prediction using Way2Drug CLC-pred.*

Lp.	Pa	Pi	Cell-line	Description	Tissue/Organ	Type
1	0.725	0.003	MES-SA	Uterine corpus sarcoma	Uterus	Sarcoma
2	0.617	0.103	A2780cisR	Cisplatin-resistant ovarian carcinoma	Ovarium	Carcinoma
3	0.431	0.070	HepG2	Hepatoblastoma	Liver	Hepatoblastoma
4	0.408	0.007	5637	Urothelial bladder carcinoma	Urinary tract	Carcinoma
5	0.391	0.093	YAPC	Pancreatic carcinoma	Pancreas	Carcinoma
6	0.352	0.030	NCI-H661	Lung carcinoma	Lung	Carcinoma
7	0.348	0.006	C8166	Leukemic T-cells	Blood	Leukemia
8	0.325	0.144	SK-MES-1	Squamous cell lung carcinoma	Lung	Carcinoma
9	0.322	0.191	SK-MEL-1	Metastatic melanoma	Skin	Melanoma
10	0.317	0.248	SK-LU-1	Adenocarcinoma	Lung	Carcinoma

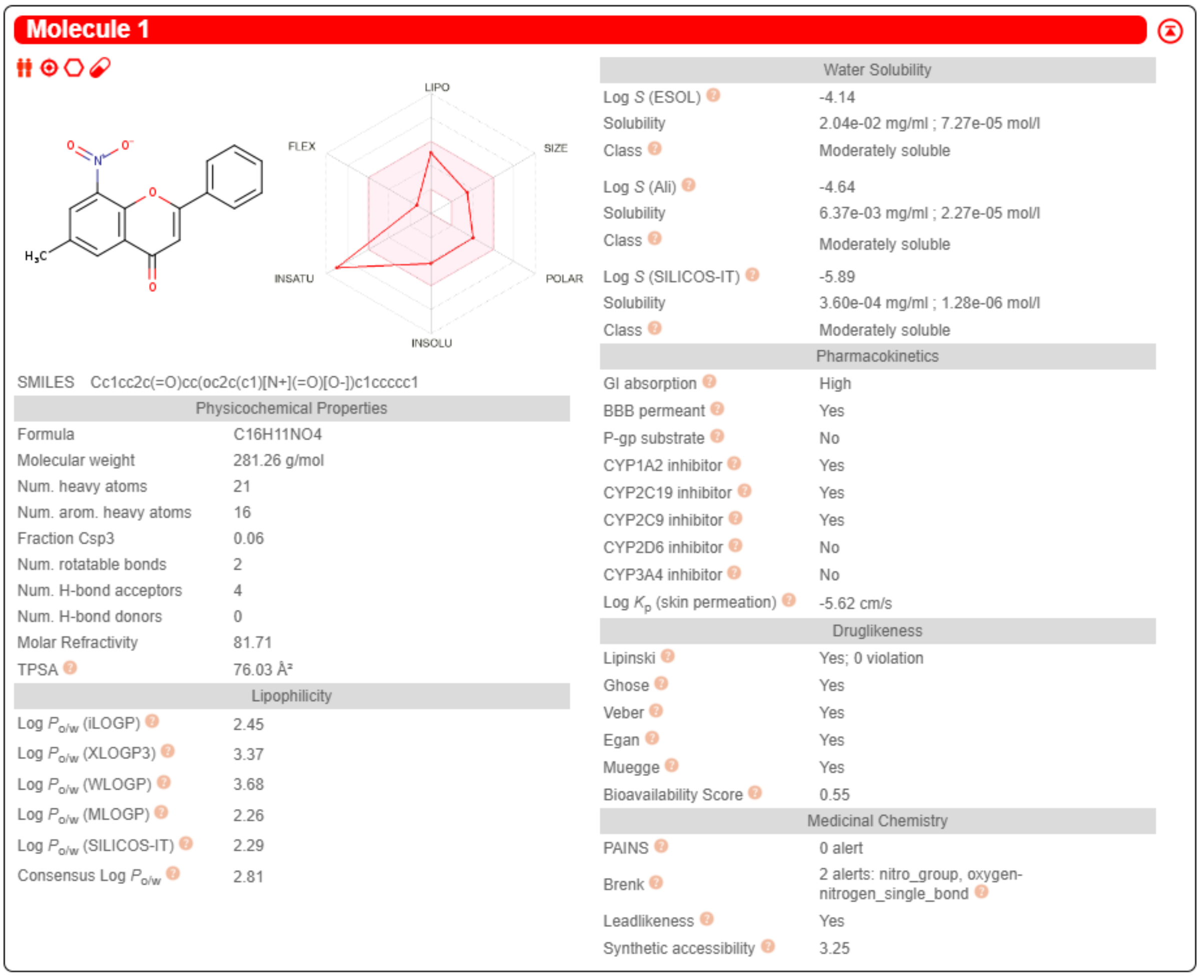

*Figure S 3. 6-Methyl-8-nitroflavone (**3**) activity prediction using SwissADME.*

Compound name: 3′-Bromo-5′-chloro-2′-hydroxychalcone
Symbol: **4**
Molecular Formula: $C_{15}H_{10}BrClO_2$
Formula Weight: 337.596
SMILE: Oc1c(cc(Cl)cc1Br)C(=O)/C=C/c1ccccc1

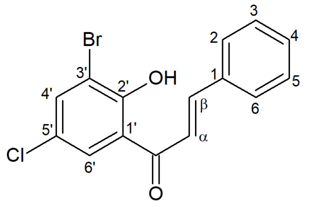

*Table S 4. 3′-Bromo-5′-chloro-2′-hydroxychalcone (**4**) cytotoxicity prediction using Way2Drug CLC-pred.*

Lp.	Pa	Pi	Cell-line	Description	Tissue/Organ	Type
1	0.716	0.062	A2780cisR	Cisplatin-resistant ovarian carcinoma	Ovarium	Carcinoma
2	0.504	0.086	MCF7	Breast carcinoma	Breast	Carcinoma
3	0.388	0.053	K562	Erythroleukemia	Haematopoietic and lymphoid tissue	Leukemia
4	0.386	0.054	TK-10	Renal carcinoma	Kidney	Carcinoma
5	0.386	0.065	SNU-475	Hepatocellular carcinoma	Liver	Carcinoma
6	0.360	0.031	RCC4	Clear cell renal cell carcinoma	Kidney	Carcinoma
7	0.352	0.021	RL	Non-Hodgkin's Lymphoma	Ascites	Lymphoma
8	0.340	0.206	DU-4475	Breast Carcinoma	Breast	Carcinoma
9	0.323	0.231	SW48	Colorectal Adenocarcinoma	Colon	Adenocarcinoma
10	0.320	0.006	KOPTK1	Childhood T acute lymphoblastic leukemia	Blood	Leukemia

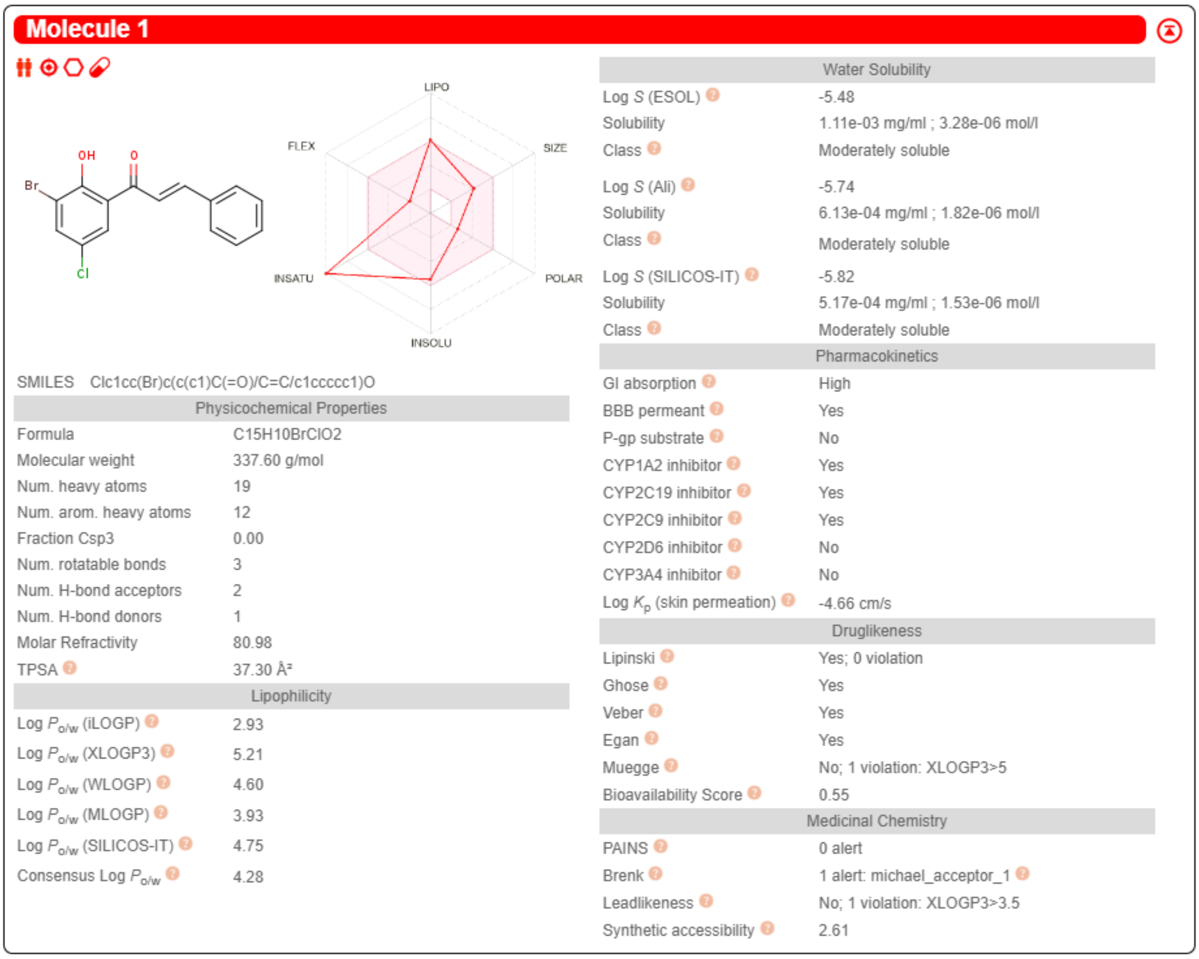

*Figure S 4. 3′-Bromo-5′-chloro-2′-hydroxychalcone (**4**) activity prediction using SwissADME.*

Compound name: 8-Bromo-6-chloroflavanone
Symbol: **5**
Molecular Formula: $C_{15}H_{10}BrClO_2$
Formula Weight: 337.596
SMILE: Clc1cc(Br)c2OC(CC(=O)c2c1)c1ccccc1

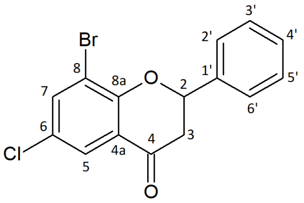

*Table S 5. 8-Bromo-6-chloroflavanone (**5**) cytotoxicity prediction using Way2Drug CLC-pred.*

Lp.	Pa	Pi	Cell-line	Description	Tissue/Organ	Type
1	0.754	0.048	A2780cisR	Cisplatin-resistant ovarian carcinoma	Ovarium	Carcinoma
2	0.373	0.068	SK-MES-1	Squamous cell lung carcinoma	Lung	Carcinoma
3	0.373	0.148	DU-4475	Breast Carcinoma	Breast	Carcinoma
4	0.365	0.116	SK-MEL-1	Metastatic melanoma	Skin	Melanoma
5	0.344	0.083	NCI-H187	Small cell lung carcinoma	Lung	Carcinoma
6	0.335	0.021	NALM-6	Adult B acute lymphoblastic leukemia	Haematopoietic and lymphoid tissue	Leukemia
7	0.302	0.294	MONO-MAC-6	Adult acute monocytic leukemia	Blood	Leukemia
8	0.269	0.045	SW1116	Colorectal Adenocarcinoma	Colon	Adenocarcinoma
9	0.257	0.031	MDA-MB-435	Amelanotic melanoma	Breast	Melanoma
10	0.255	0.147	MKN-74	Gastric tubular adenocarcinoma	Stomach	Adenocarcinoma

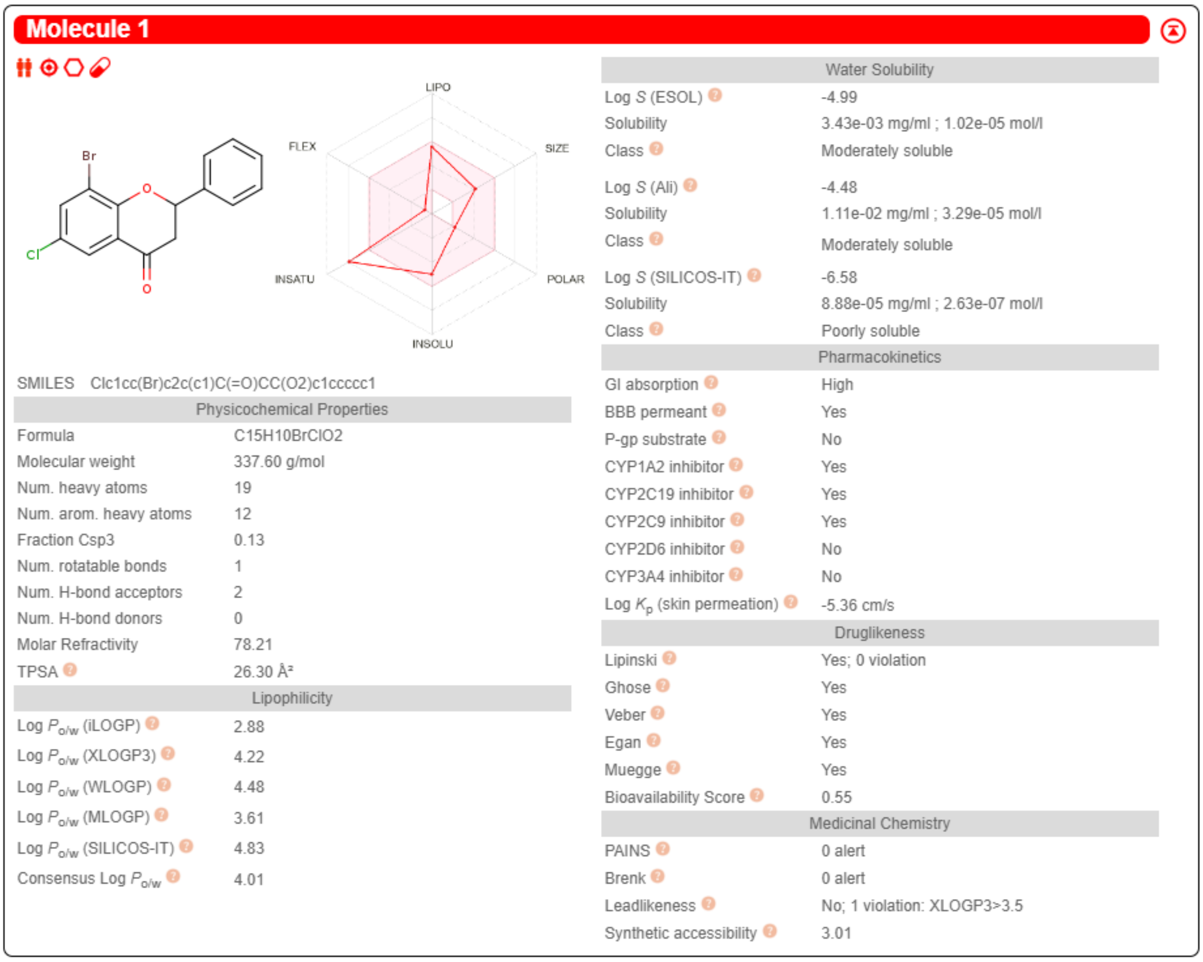

*Figure S 5. 8-Bromo-6-chloroflavanone (**5**) activity prediction using SwissADME.*

Compound name: 8-Bromo-6-chloroflavone
Symbol: **6**
Molecular Formula: $C_{15}H_8BrClO_2$
Formula Weight: 335.579
SMILE: Clc1cc(Br)c2OC(=CC(=O)c2c1)c1ccccc1

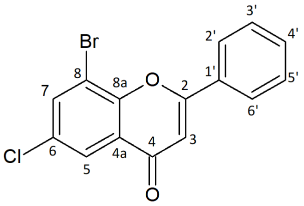

*Table S 6. 8-Bromo-6-chloroflavone (**6**) cytotoxicity prediction using Way2Drug CLC-pred.*

Lp.	Pa	Pi	Cell-line	Description	Tissue/Organ	Type
1	0.609	0.107	A2780cisR	Cisplatin-resistant ovarian carcinoma	Ovarium	Carcinoma
2	0.426	0.050	SK-MEL-1	Metastatic melanoma	Skin	Melanoma
3	0.381	0.058	SK-MES-1	Squamous cell lung carcinoma	Lung	Carcinoma
4	0.374	0.147	DU-4475	Breast Carcinoma	Breast	Carcinoma
5	0.354	0.204	MONO-MAC-6	Adult acute monocytic leukemia	Blood	Leukemia
6	0.336	0.115	COR-L23	Lung large cell carcinoma	Lung	Carcinoma
7	0.320	0.044	NALM-6	Adult B acute lymphoblastic leukemia	Haematopoietic and lymphoid tissue	Leukemia
8	0.317	0.077	NCI-H661	Lung carcinoma	Lung	Carcinoma
9	0.306	0.090	HuP-T3	Pancreatic adenocarcinoma	Pancreas	Adenocarcinoma
10	0.287	0.281	CA46	Burkitts Lymphoma	Blood	Lymphoma

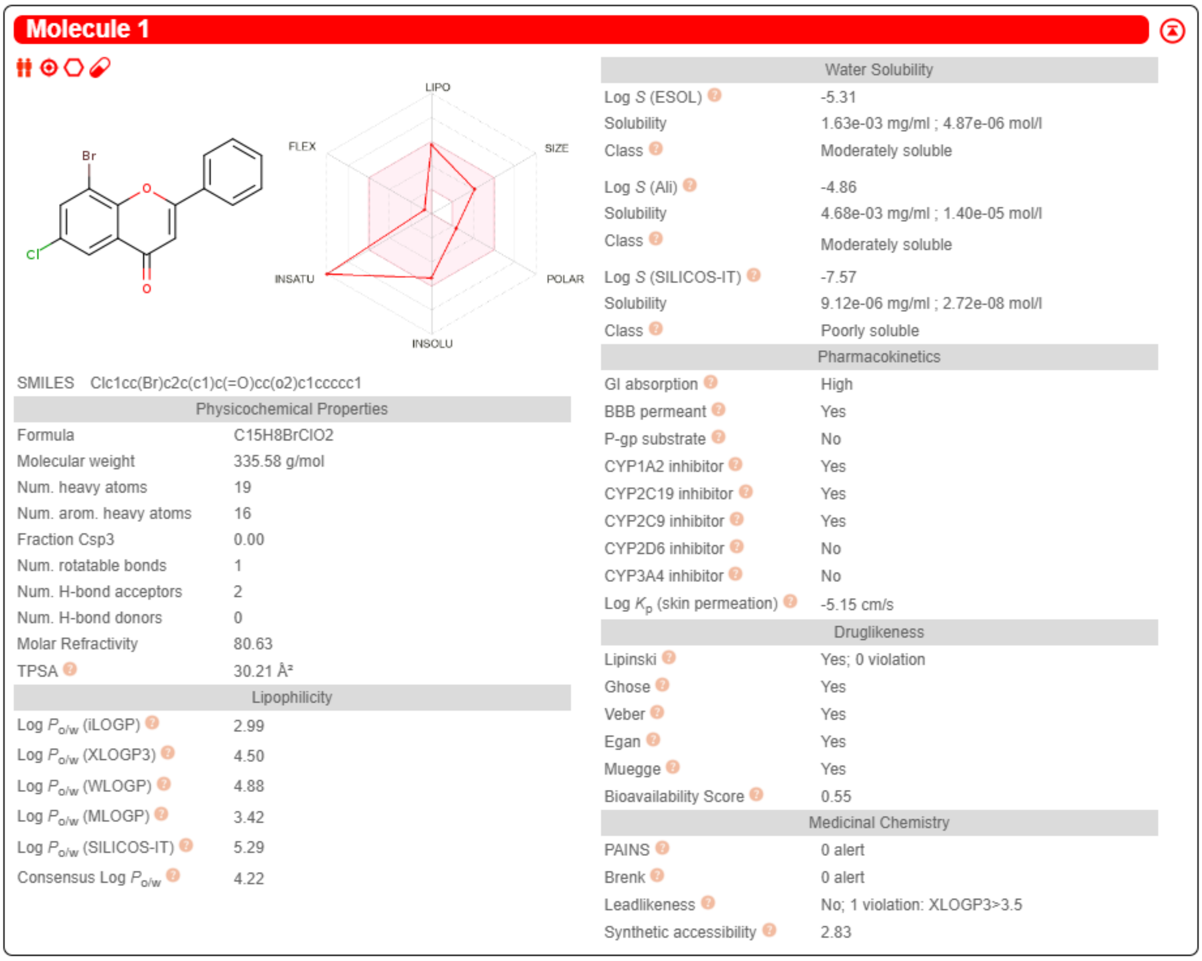

*Figure S 6. 8-Bromo-6-chloroflavone (**6**) activity prediction using SwissADME.*

Compound name: 8-Bromo-6-chloroflavone 4′-*O*-*β*-D-(4″-*O*-methyl)-glucopyranoside
Symbol: **6a**
Molecular Formula: $C_{22}H_{20}BrClO_8$
Formula Weight: 527.746
SMILE:
O=C1C=C(Oc2c1cc(Cl)cc2Br)c1ccc(cc1)O[C@@H]1OC(CO)[C@@H](OC)C(O)C1O

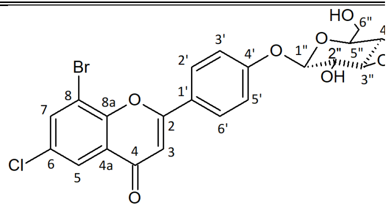

*Table S 7. 8-Bromo-6-chloroflavone 4′-O-β-D-(4″-O-methyl)-glucopyranoside (**6a**) cytotoxicity prediction using Way2Drug CLC-pred.*

Lp.	Pa	Pi	Cell-line	Description	Tissue/Organ	Type
1	0.500	0.015	MRC5	Embryonic lung fibroblast	Lung	Normal
2	0.497	0.020	SK-MEL-1	Metastatic melanoma	Skin	Melanoma
3	0.489	0.036	DU-4475	Breast Carcinoma	Breast	Carcinoma
4	0.488	0.044	HL-60	Promyeloblast leukemia	Haematopoietic and lymphoid tissue	Leukemia
5	0.481	0.013	SW1990	Pancreatic adenocarcinoma	Pancreas	Adenocarcinoma
6	0.458	0.013	HEL299	Fibroblasts	Lung	Normal
7	0.447	0.014	COR-L23	Lung large cell carcinoma	Lung	Carcinoma
8	0.411	0.158	SK-LU-1	Adenocarcinoma	Lung	Carcinoma
9	0.384	0.027	H9	T-lymphoid	Haematopoietic and lymphoid tissue	Leukemia
10	0.380	0.059	SK-MES-1	Squamous cell lung carcinoma	Lung	Carcinoma

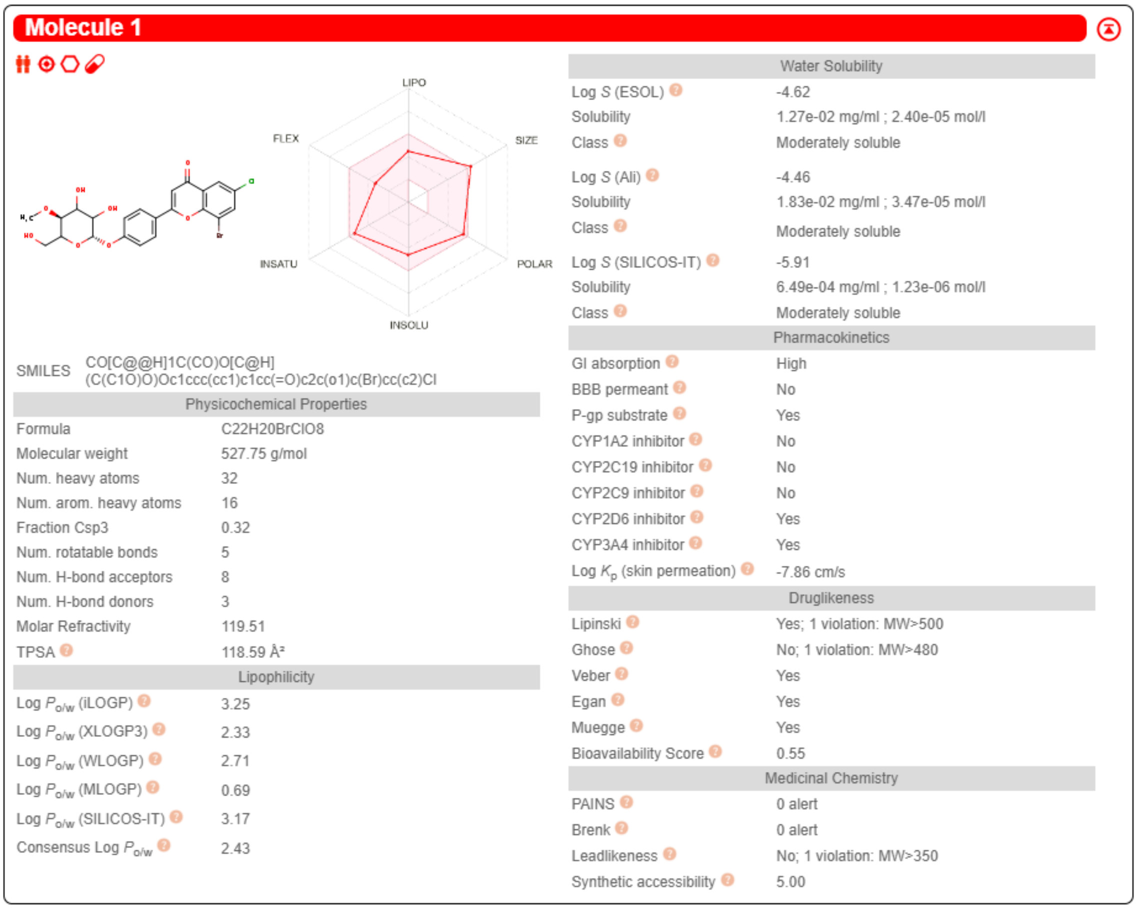

*Figure S 7. 8-Bromo-6-chloroflavone 4′-O-β-D-(4″-O-methyl)-glucopyranoside (**6a**) activity prediction using SwissADME.*

Compound name: 5′-Chloro-2′-hydroxy-3′-nitrochalcone
Symbol: **7**
Molecular Formula: $C_{15}H_{10}ClNO_4$
Formula Weight: 303.697
SMILE: O=[N+]([O-])c1cc(Cl)cc(c1O)C(=O)/C=C/c1ccccc1

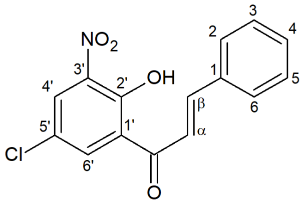

*Table S 8. 5′-Chloro-2′-hydroxy-3′-nitrochalcone (**7**) cytotoxicity prediction using Way2Drug CLC-pred.*

Lp.	Pa	Pi	Cell-line	Description	Tissue/Organ	Type
1	0.647	0.091	A2780cisR	Cisplatin-resistant ovarian carcinoma	Ovarium	Carcinoma
2	0.484	0.030	K562	Erythroleukemia	Haematopoietic and lymphoid tissue	Leukemia
3	0.484	0.054	HepG2	Hepatoblastoma	Liver	Hepatoblastoma
4	0.435	0.024	HEK293	Embryonic kidney fibroblast	Kidney	Normal
5	0.373	0.072	CAL-51	Breast carcinoma	Breast	Carcinoma
6	0.371	0.081	SNU-475	Hepatocellular carcinoma	Liver	Carcinoma
7	0.365	0.126	YAPC	Pancreatic carcinoma	Pancreas	Carcinoma
8	0.347	0.023	RL	Non-Hodgkin's Lymphoma	Ascites	Lymphoma
9	0.334	0.041	RCC4	Clear cell renal cell carcinoma	Kidney	Carcinoma
10	0.333	0.189	NCI-H441	Papillary adenocarcinoma	Lung	Adenocarcinoma

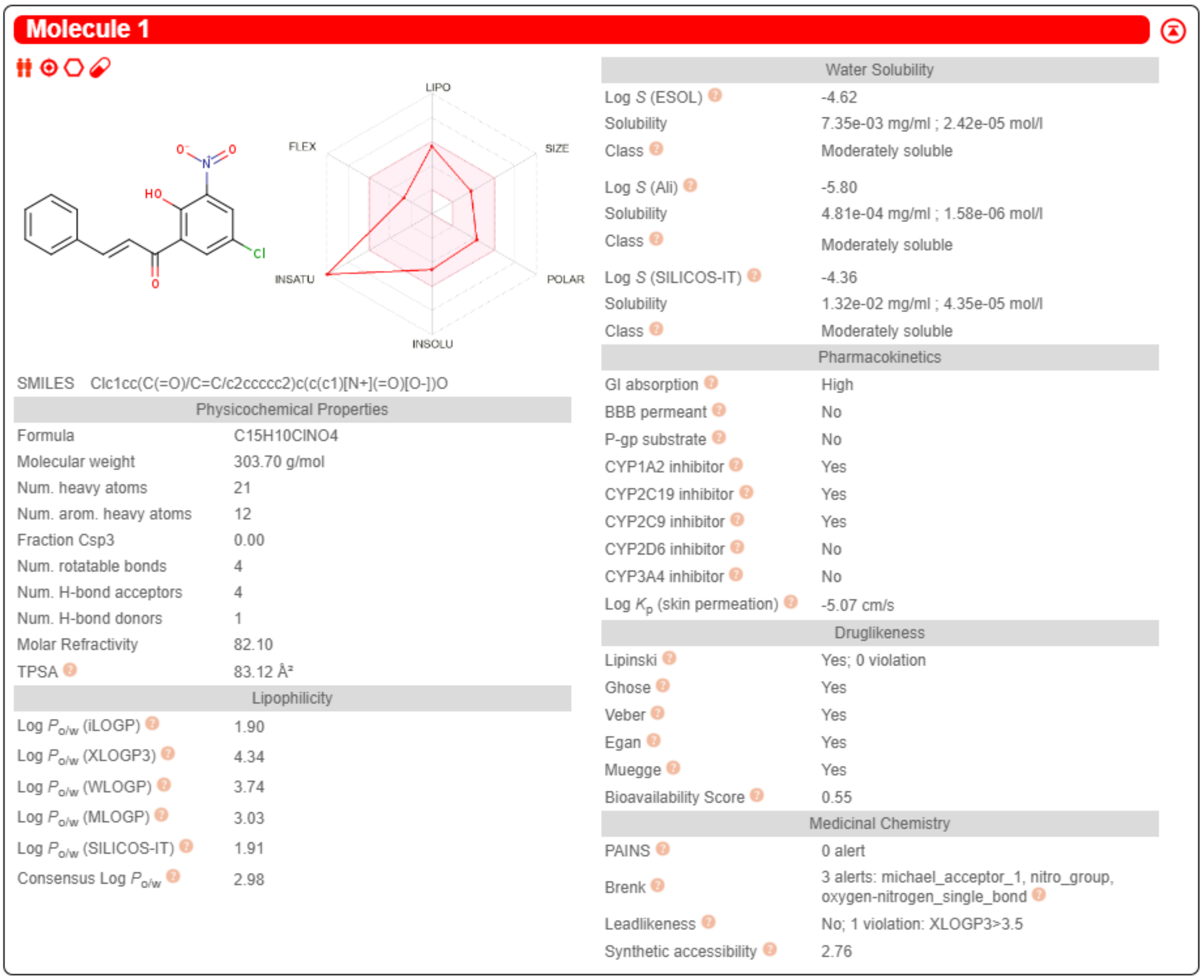

*Figure S 8. 5′-Chloro-2′-hydroxy-3′-nitrochalcone (**7**) activity prediction using SwissADME.*

Compound name: 6-Chloro-8-nitroflavanone
Symbol: **8**
Molecular Formula: $C_{15}H_{10}ClNO_4$
Formula Weight: 303.697
SMILE: [O-][N+](=O)c1cc(Cl)cc2c1OC(CC2=O)c1ccccc1

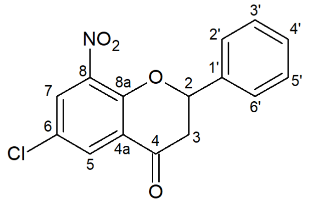

Table S 9. 6-Chloro-8-nitroflavanone (8) cytotoxicity prediction using Way2Drug CLC-pred.

Lp.	Pa	Pi	Cell-line	Description	Tissue/Organ	Type
1	0.693	0.072	A2780cisR	Cisplatin-resistant ovarian carcinoma	Ovarium	Carcinoma
2	0.573	0.004	MES-SA	Uterine corpus sarcoma	Uterus	Sarcoma
3	0.358	0.069	NCI-H187	Small cell lung carcinoma	Lung	Carcinoma
4	0.337	0.019	NALM-6	Adult B acute lymphoblastic leukemia	Haematopoietic and lymphoid tissue	Leukemia
5	0.335	0.043	5637	Urothelial bladder carcinoma	Urinary tract	Carcinoma
6	0.319	0.101	HCC1806	Acantholytic Squamous Cell Carcinoma	Breast	Carcinoma
7	0.302	0.211	YAPC	Pancreatic carcinoma	Pancreas	Carcinoma
8	0.290	0.066	C8166	Leukemic T-cells	Blood	Leukemia
9	0.290	0.202	SK-MES-1	Squamous cell lung carcinoma	Lung	Carcinoma
10	0.265	0.129	MKN-74	Gastric tubular adenocarcinoma	Stomach	Adenocarcinoma

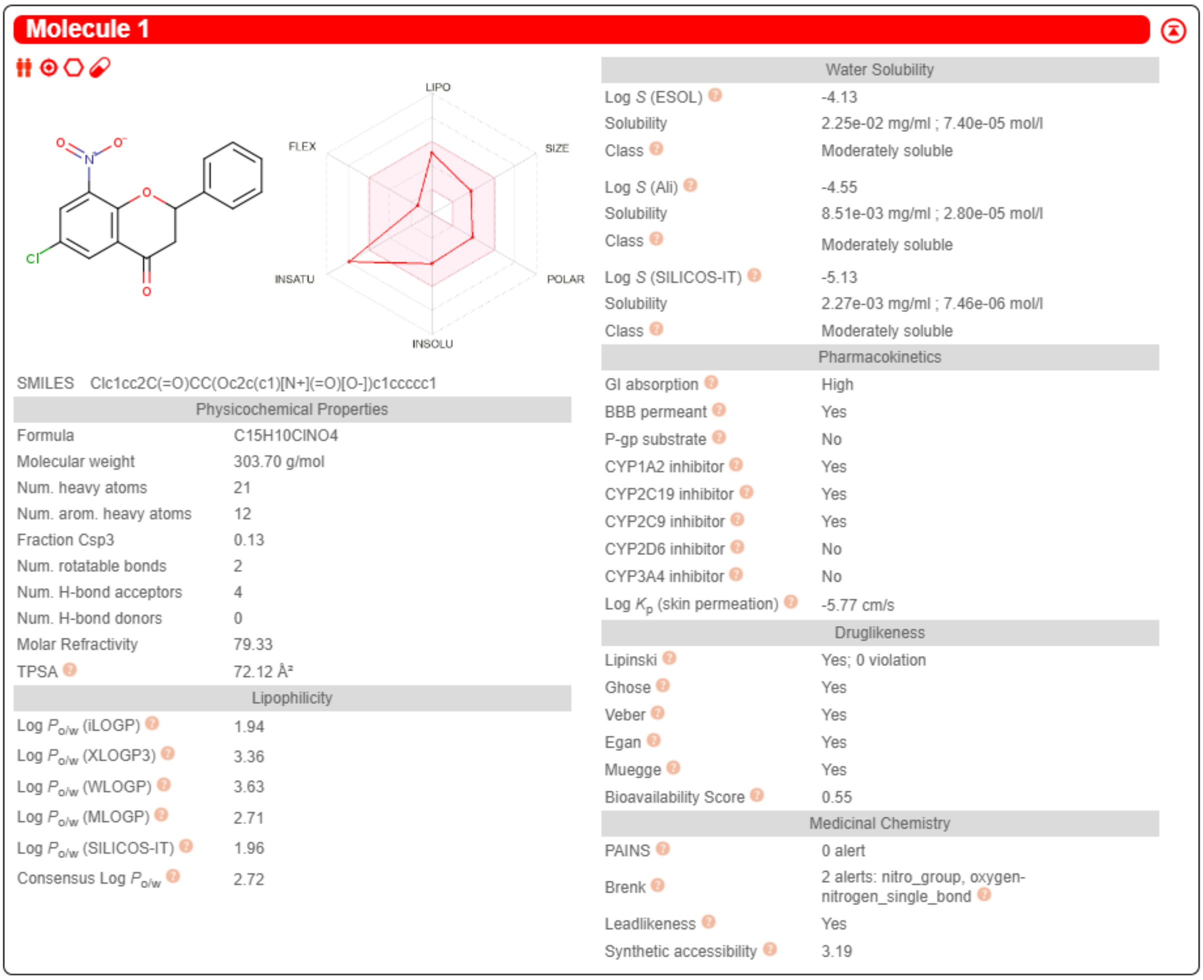

Figure S 9. 6-Chloro-8-nitroflavanone (8) activity prediction using SwissADME.

Compound name: 6-Chloro-8-nitroflavone
Symbol: **9**
Molecular Formula: $C_{15}H_8ClNO_4$
Formula Weight: 301.681
SMILE: [O-][N+](=O)c1cc(Cl)cc2c1OC(=CC2=O)c1ccccc1

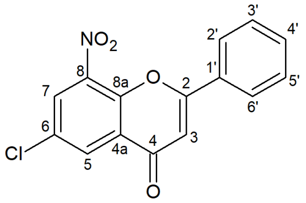

*Table S 10. 6-Chloro-8-nitroflavone (**9**) cytotoxicity prediction using Way2Drug CLC-pred.*

Lp.	Pa	Pi	Cell-line	Description	Tissue/Organ	Type
1	0.704	0.003	MES-SA	Uterine corpus sarcoma	Uterus	Sarcoma
2	0.536	0.043	HepG2	Hepatoblastoma	Liver	Hepatoblastoma
3	0.523	0.147	A2780cisR	Cisplatin-resistant ovarian carcinoma	Ovarium	Carcinoma
4	0.400	0.008	5637	Urothelial bladder carcinoma	Urinary tract	Carcinoma
5	0.346	0.218	MONO-MAC-6	Adult acute monocytic leukemia	Blood	Leukemia
6	0.339	0.044	NCI-H661	Lung carcinoma	Lung	Carcinoma
7	0.337	0.008	C8166	Leukemic T-cells	Blood	Leukemia
8	0.324	0.035	NALM-6	Adult B acute lymphoblastic leukemia	Haematopoietic and lymphoid tissue	Leukemia
9	0.318	0.192	CA46	Burkitts Lymphoma	Blood	Lymphoma
10	0.305	0.134	HCC1806	Acantholytic Squamous Cell Carcinoma	Breast	Carcinoma

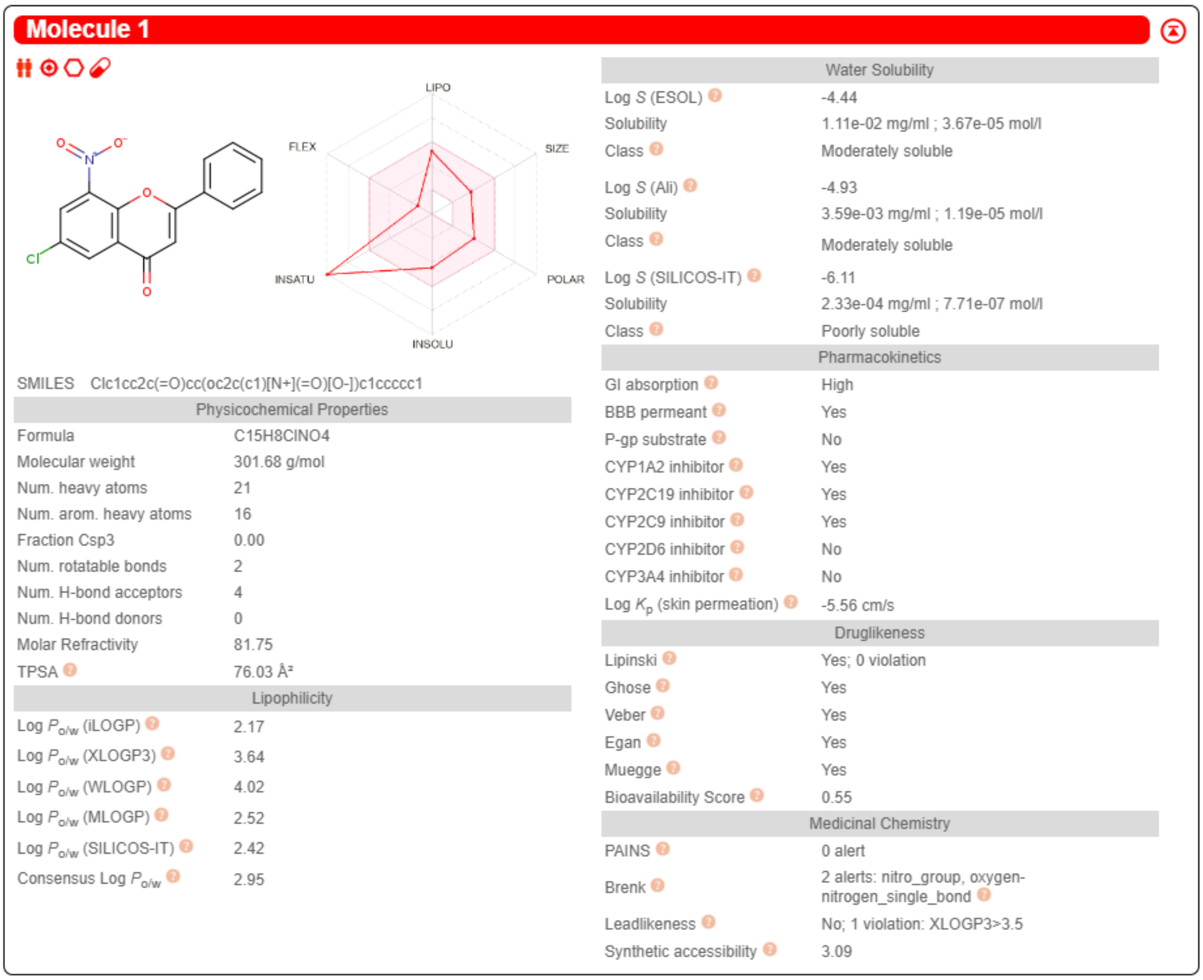

*Figure S 10. 6-Chloro-8-nitroflavone (**9**) activity prediction using SwissADME.*

Compound name: 5′-Bromo-2′-hydroxy-3′-nitrochalcone
Symbol: **10**
Molecular Formula: $C_{15}H_{10}BrNO_4$
Formula Weight: 348.148
SMILE: O=[N+]([O-])c1cc(Br)cc(c1O)C(=O)/C=C/c1ccccc1

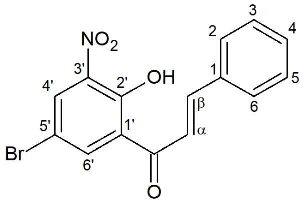

*Table S 11. 5′-Bromo-2′-hydroxy-3′-nitrochalcone (**10**) cytotoxicity prediction using Way2Drug CLC-pred.*

Lp.	Pi	Pa	Cell-line	Description	Tissue/Organ	Type
1	0.647	0.091	A2780cisR	Cisplatin-resistant ovarian carcinoma	Ovarium	Carcinoma
2	0.503	0.026	K562	Erythroleukemia	Haematopoietic and lymphoid tissue	Leukemia
3	0.475	0.057	HepG2	Hepatoblastoma	Liver	Hepatoblastoma
4	0.436	0.024	HEK293	Embryonic kidney fibroblast	Kidney	Normal
5	0.435	0.120	MCF7	Breast carcinoma	Breast	Carcinoma
6	0.371	0.081	SNU-475	Hepatocellular carcinoma	Liver	Carcinoma
7	0.356	0.136	YAPC	Pancreatic carcinoma	Pancreas	Carcinoma
8	0.355	0.020	RL	Non-Hodgkin's Lymphoma	Ascites	Lymphoma
9	0.334	0.041	RCC4	Clear cell renal cell carcinoma	Kidney	Carcinoma
10	0.330	0.149	CAL-51	Breast carcinoma	Breast	Carcinoma

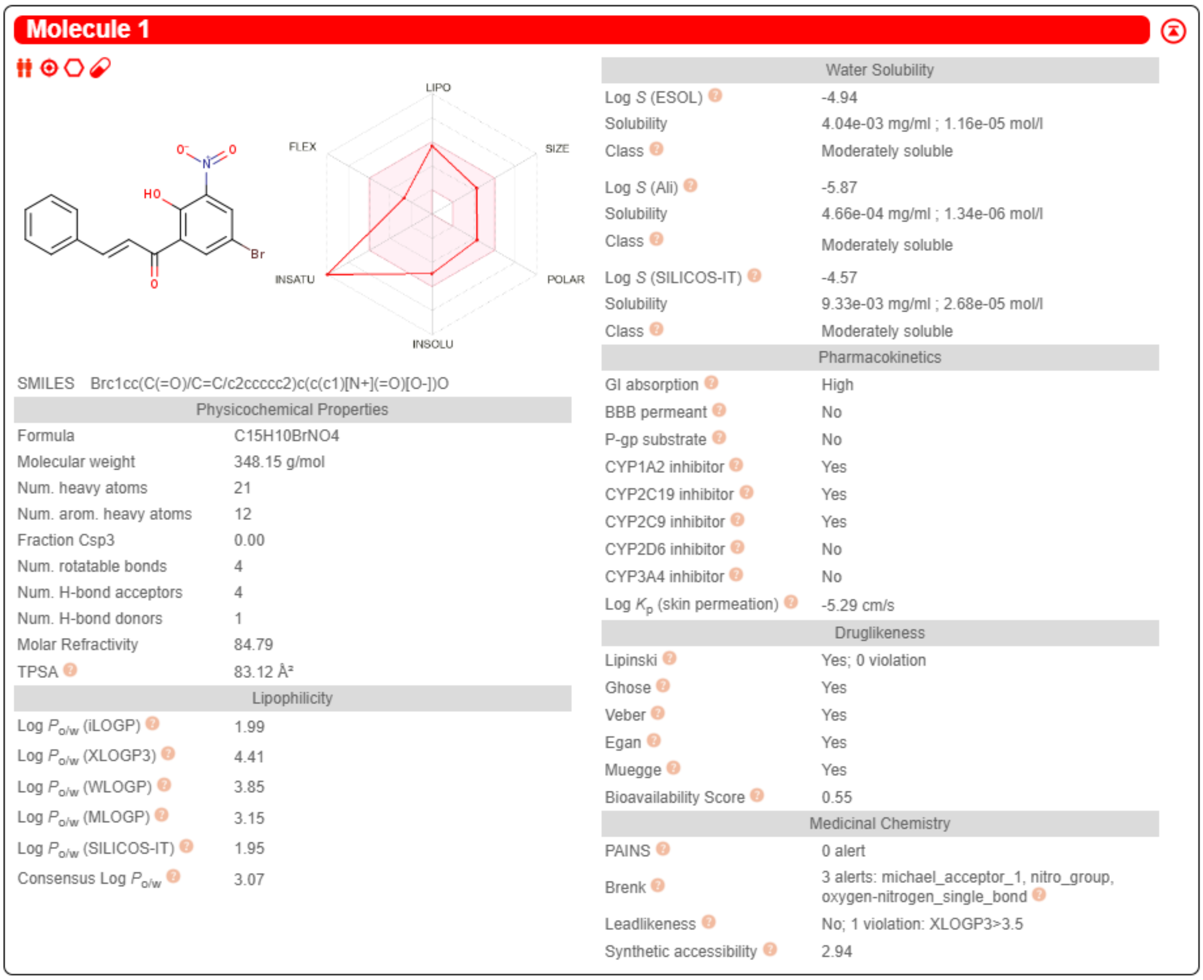

*Figure S 11. 5′-Bromo-2′-hydroxy-3′-nitrochalcone (**10**) activity prediction using SwissADME.*

Compound name: 6-Bromo-8-nitroflavanone
Symbol: **11**
Molecular Formula: $C_{15}H_{10}BrNO_4$
Formula Weight: 348.148
SMILE: [O-][N+](=O)c1cc(Br)cc2c1OC(CC2=O)c1ccccc1

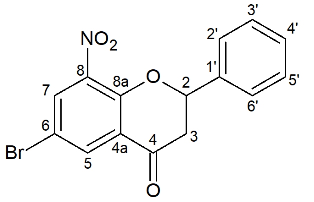

*Table S 12. 6-Bromo-8-nitroflavanone (**11**) cytotoxicity prediction using Way2Drug CLC-pred.*

Lp.	Pa	Pi	Cell-line	Description	Tissue/Organ	Type
1	0.693	0.072	A2780cisR	Cisplatin-resistant ovarian carcinoma	Ovarium	Carcinoma
2	0.555	0.004	MES-SA	Uterine corpus sarcoma	Uterus	Sarcoma
3	0.343	0.084	NCI-H187	Small cell lung carcinoma	Lung	Carcinoma
4	0.335	0.021	NALM-6	Adult B acute lymphoblastic leukemia	Haematopoietic and lymphoid tissue	Leukemia
5	0.325	0.190	MCF7	Breast carcinoma	Breast	Carcinoma
6	0.291	0.230	YAPC	Pancreatic carcinoma	Pancreas	Carcinoma
7	0.270	0.175	5637	Urothelial bladder carcinoma	Urinary tract	Carcinoma
8	0.268	0.130	C8166	Leukemic T-cells	Blood	Leukemia
9	0.235	0.127	NCI-H295R	Adrenal cortex carcinoma	Adrenal cortex	Carcinoma
10	0.214	0.142	SW1116	Colorectal Adenocarcinoma	Colon	Adenocarcinoma

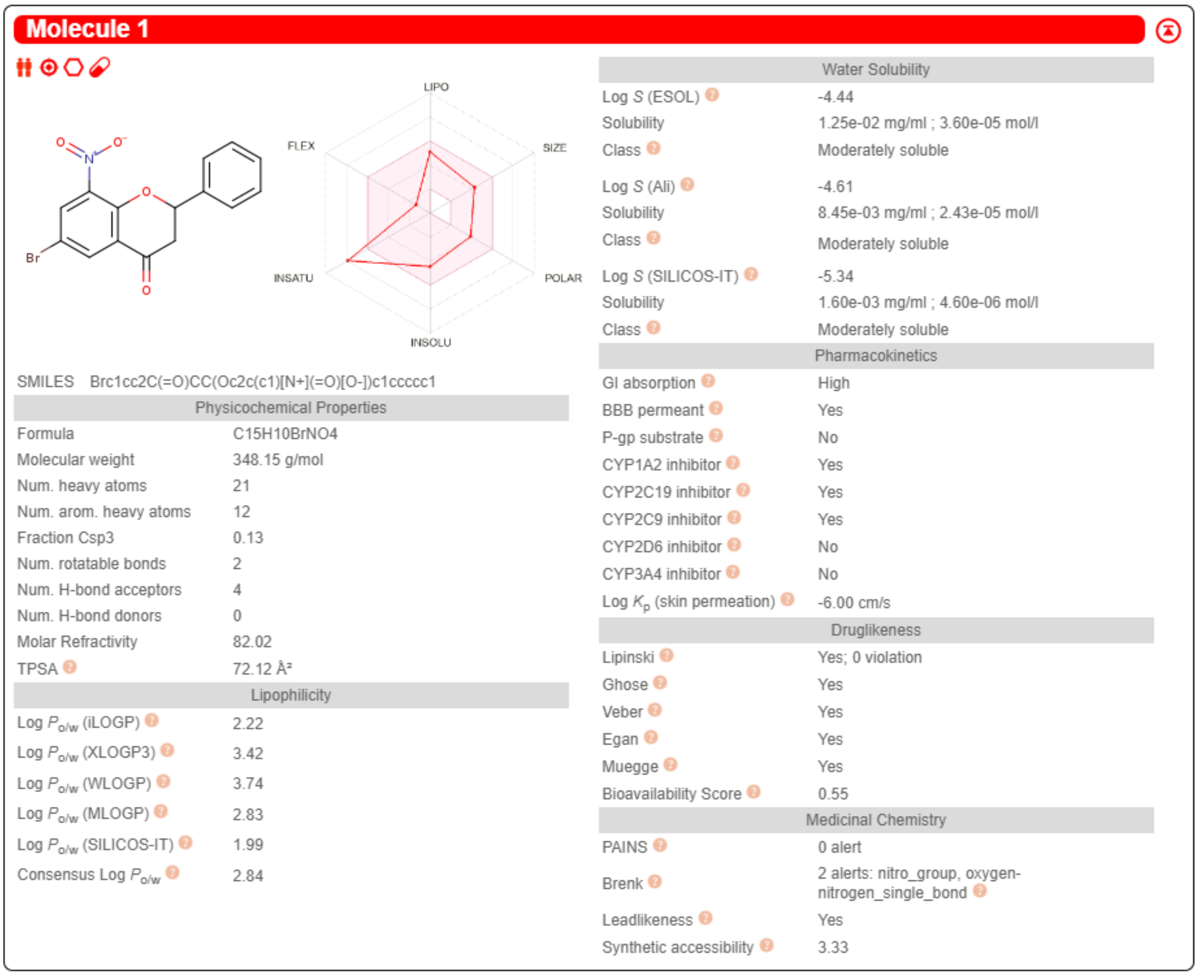

*Figure S 12. 6-Bromo-8-nitroflavanone (**11**) activity prediction using SwissADME.*

Compound name: 6-Bromo-8-nitroflavone
Symbol: **12**
Molecular Formula: $C_{15}H_8BrNO_4$
Formula Weight: 346.132
SMILE: [O-][N+](=O)c1cc(Br)cc2c1OC(=CC2=O)c1ccccc1

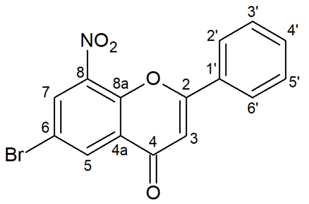

*Table S 13. 6-Bromo-8-nitroflavone (**12**) cytotoxicity prediction using Way2Drug CLC-pred.*

Lp.	Pa	Pi	Cell-line	Description	Tissue/Organ	Type
1	0.687	0.003	MES-SA	Uterine corpus sarcoma	Uterus	Sarcoma
2	0.606	0.031	HepG2	Hepatoblastoma	Liver	Hepatoblastoma
3	0.523	0.147	A2780cisR	Cisplatin-resistant ovarian carcinoma	Ovarium	Carcinoma
4	0.368	0.161	MCF7	Breast carcinoma	Breast	Carcinoma
5	0.362	0.020	5637	Urothelial bladder carcinoma	Urinary tract	Carcinoma
6	0.325	0.014	C8166	Leukemic T-cells	Blood	Leukemia
7	0.321	0.041	NALM-6	Adult B acute lymphoblastic leukemia	Haematopoietic and lymphoid tissue	Leukemia
8	0.320	0.072	NCI-H661	Lung carcinoma	Lung	Carcinoma
9	0.306	0.225	SK-MEL-1	Metastatic melanoma	Skin	Melanoma
10	0.280	0.039	G-361	Melanoma	Skin	Melanoma

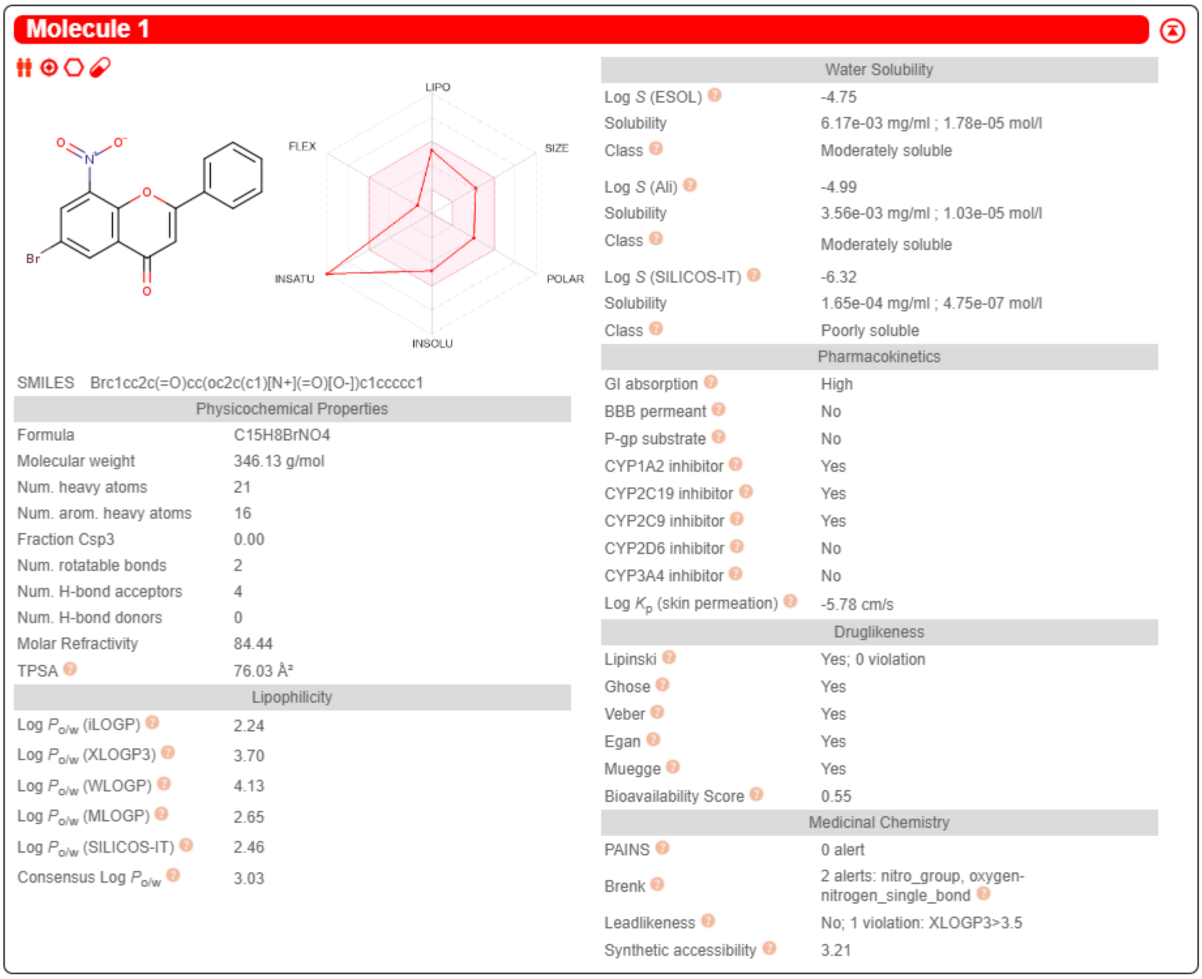

*Figure S 13. 6-Bromo-8-nitroflavone (**12**) activity prediction using SwissADME*

Table S 14. Unequal variances t-test with Holm–Bonferroni correction for E. faecalis.

Probe	*E. faecalis* + DMSO	*E. faecalis* + (1) 0.1%	*E. faecalis* + (1) 0.05%	*E. faecalis* + (4) 0.1%	*E. faecalis* + (4) 0.05%	*E. faecalis* + (2) 0.1%	*E. faecalis* + (2) 0.05%	*E. faecalis* + (5) 0.1%	*E. faecalis* + (5) 0.05%	*E. faecalis* + (3) 0.1%	*E. faecalis* + (3) 0.05%	*E. faecalis* + (6) 0.1%	*E. faecalis* + (6) 0.05%	*E. faecalis* + (6a) 0.1%	*E. faecalis* + (6a) 0.05%
P-value	0.026	0.019	0.040	0.000	0.047	0.002	0.001	0.001	0.001	0.000	0.045	0.044	0.029	0.022	0.041
	*	*	*	***	*	**	***	***	***	***	*	*	*	*	*

P-value <0.05 mark as significant.

Table S 15. Unequal variances t-test with Holm–Bonferroni correction for S. aureus.

Probe	*S. aureus* + DMSO	*S. aureus* + (1) 0.1%	*S. aureus* + (1) 0.05%	*S. aureus* + (4) 0.1%	*S. aureus* + (4) 0.05%	*S. aureus* + (2) 0.1%	*S. aureus* + (2) 0.05%	*S. aureus* + (5) 0.1%	*S. aureus* + (5) 0.05%	*S. aureus* + (3) 0.1%	*S. aureus* + (3) 0.05%	*S. aureus* + (6) 0.1%	*S. aureus* + (6) 0.05%	*S. aureus* + (6a) 0.1%	*S. aureus* + (6a) 0.05%
P-value	0.139	0.006	0.003	0.020	0.054	0.020	0.029	0.117	0.098	0.061	0.061	0.002	0.114	0.051	0.286
		**	**	*		*	*					**			

P-value <0.05 mark as significant.

Table S 16. Unequal variances t-test with Holm–Bonferroni correction for E. coli.

Probe	*E. coli* + DMSO	*E. coli* + (1) 0.1%	*E. coli* + (1) 0.05%	*E. coli* + (4) 0.1%	*E. coli* + (4) 0.05%	*E. coli* + (2) 0.1%	*E. coli* + (2) 0.05%	*E. coli* + (5) 0.1%	*E. coli* + (5) 0.05%	*E. coli* + (3) 0.1%	*E. coli* + (3) 0.05%	*E. coli* + (6) 0.1%	*E. coli* + (6) 0.05%	*E. coli* + (6a) 0.1%	*E. coli* + (6a) 0.05%
P-value	0.401	0.001	0.111	0.002	0.003	0.000	0.068	0.377	0.242	0.188	0.115	0.008	0.001	0.930	0.625
		***		**	**	***						**	***		

P-value <0.05 mark as significant.

Table S 17. Unequal variances t-test with Holm–Bonferroni correction for C. albicans.

Probe	*C. albicans* + DMSO	*C. albicans* + (1) 0.1%	*C. albicans* + (1) 0.05%	*C. albicans* + (4) 0.1%	*C. albicans* + (4) 0.05%	*C. albicans* + (2) 0.1%	*C. albicans* + (2) 0.05%	*C. albicans* + (5) 0.1%	*C. albicans* + (5) 0.05%	*C. albicans* + (3) 0.1%	*C. albicans* + (3) 0.05%	*C. albicans* + (6) 0.1%	*C. albicans* + (6) 0.05%	*C. albicans* + (6a) 0.1%	*C. albicans* + (6a) 0.05%
P-value	0.074	0.007	0.105	0.000	0.117	0.000	0.265	0.075	0.174	0.003	0.025	0.068	0.508	0.328	0.008
		**		***		***				**	*				**

P-value <0.05 mark as significant.

Table S 18. Unequal variances t-test with Holm–Bonferroni correction for L. acidophilus.

Probe	*L. acidophilus* + DMSO	*L. acidophilus* + (1) 0.1%	*L. acidophilus* + (1) 0.05%	*L. acidophilus* + (4) 0.1%	*L. acidophilus* + (4) 0.05%	*L. acidophilus* + (2) 0.1%	*L. acidophilus* + (2) 0.05%	*L. acidophilus* + (5) 0.1%	*L. acidophilus* + (5) 0.05%	*L. acidophilus* + (3) 0.1%	*L. acidophilus* + (3) 0.05%	*L. acidophilus* + (6) 0.1%	*L. acidophilus* + (6) 0.05%	*L. acidophilus* + (6a) 0.1%	*L. acidophilus* + (6a) 0.05%
P-value	0.042	0.002	0.052	0.004	0.049	0.066	0.088	0.077	0.049	0.111	0.056	0.095	0.037	0.018	0.259
	*	**		**	*				*				*	*	

P-value <0.05 mark as significant.

Table S 19. Unequal variances t-test with Holm–Bonferroni correction for L. casei.

Probe	*L. casei* + DMSO	*L. casei* + (1) 0.1%	*L. casei* + (1) 0.05%	*L. casei* + (4) 0.1%	*L. casei* + (4) 0.05%	*L. casei* + (2) 0.1%	*L. casei* + (2) 0.05%	*L. casei* + (5) 0.1%	*L. casei* + (5) 0.05%	*L. casei* + (3) 0.1%	*L. casei* + (3) 0.05%	*L. casei* + (6) 0.1%	*L. casei* + (6) 0.05%	*L. casei* + (6a) 0.1%	*L. casei* + (6a) 0.05%
P-value	1	0.840	1	0.039	0.819	0.844	1	0.868	1	0.564	0.979	0.767	1	0.527	0.463
				*											

P-value <0.05 mark as significant.

Table S 20. Unequal variances t-test with Holm–Bonferroni correction for L. plantarum.

Probe	*L. plantarum* + DMSO	*L. plantarum* + (1) 0.1%	*L. plantarum* + (1) 0.05%	*L. plantarum* + (4) 0.1%	*L. plantarum* + (4) 0.05%	*L. plantarum* + (2) 0.1%	*L. plantarum* + (2) 0.05%	*L. plantarum* + (5) 0.1%	*L. plantarum* + (5) 0.05%	*L. plantarum* + (3) 0.1%	*L. plantarum* + (3) 0.05%	*L. plantarum* + (6) 0.1%	*L. plantarum* + (6) 0.05%	*L. plantarum* + (6a) 0.1%	*L. plantarum* + (6a) 0.05%
P-value	0.974	0.582	1	0.512	0.853	0.810	0.128	0.906	0.155	0.985	0.988	0.812	0.892	0.223	0.999

P-value <0.05 mark as significant.

Table S 21. Unequal variances t-test with Holm–Bonferroni correction for P. pentosaceus.

Probe	*P. pentosaceus* + DMSO	*P. pentosaceus* + (1) 0.1%	*P. pentosaceus* + (1) 0.05%	*P. pentosaceus* + (4) 0.1%	*P. pentosaceus* + (4) 0.05%	*P. pentosaceus* + (2) 0.1%	*P. pentosaceus* + (2) 0.05%	*P. pentosaceus* + (5) 0.1%	*P. pentosaceus* + (5) 0.05%	*P. pentosaceus* + (3) 0.1%	*P. pentosaceus* + (3) 0.05%	*P. pentosaceus* + (6) 0.1%	*P. pentosaceus* + (6) 0.05%	*P. pentosaceus* + (6a) 0.1%	*P. pentosaceus* + (6a) 0.05%
P-value	0.109	0.984	1.553	0.125	0.553	1	0.768	0.790	1	0.902	1.642	0.314	0.351	1	1

P-value <0.05 mark as significant.

Table S 22. One-sample Student's t-test with Holm–Bonferroni correction for digestion in vitro studies.

Compound	Origin of the intestinal microbiome*	Total numer of microorganisms	*Bifidobacterium* sp.	*Lactobacillus* sp.	Nonpathogenic *E.coli*	*Enterococcus* sp.	*Clostridium* sp.	*E.coli*	Proteolytic bacteria	Yeast-like fungi
(**3**)	1M	0.523	0.595	0.321	0.186	0.307	0.296	0.296	0.176	absence
	2M	0.397	0.032 *	0.276	0.023 *	0.238	0.005 **	0.038 *	0.010 **	absence
	3M	0.980	0.392	0.675	0.601	0.617	1	0.668	0.072	absence
	4M	1	1	1	0.868	0.852	1	1	0.929	absence
	5M	0.575	0.730	0.089	0.096	0.475	0.000 ***	0.063	1	absence
(**6**)	1M	0.414	1	1	0.301	0.337	1	0.156	0.320	absence
	2M	0.791	0.048 *	0.642	0.557	0.004 **	0.126	0.546	0.288	1
	3M	0.657	1	0.374	1	1	0.998	1	absence	0.015 *
	4M	0.505	0.972	1	0.576	0.598	0.520	0.277	absence	absence
	5M	1	0.413	0.276	0.003 **	1	0.001 ***	0.019 *	0.446	absence
(**9**)	1M	0.138	0.796	1	0.308	0.268	0.306	1	1	absence
	2M	0.137	0.466	0.353	0.799	1	0.019 *	0.052	0.140	0.207
	3M	0.182	1	1	1	1	1	1	absence	1
	4M	1	1	1	1	0.940	1	0.701	absence	absence
	5M	0.594	0.799	0.557	1	0.537	0.416	0.277	0.822	absence
(**12**)	1M	1	1	1	1	0.989	0.009	1	0.221	absence
	2M	0.398	0.966	1	1	0.997	0.287	0.842	1	0.422
	3M	1	1	0.064	1	1	1	1	0.756	0.244
	4M	0.374	1	1	1	1	0.488	1	1	1
	5M	1	1	1	1	1	1	1	1	absence

*1M - Microbiome of healthy people, 2M - Microbiome of people over 75 y.o., 3M - Microbiome of people after antibiotic therapy, 4M - Microbiome of people after chemotherapy, 5M - Microbiome of obese people. P-value <0.05 mark as significant.
